# “Purple Rain, Purple Rain”: RSM Optimization and Bioactivity Assessment of Rosmarinic Acid-Rich *Salvia verticillata* L. Ethanolic Root Extract

**DOI:** 10.3390/plants15162473

**Published:** 2026-08-14

**Authors:** Nevena R. Mihailović, Nikola Z. Srećković, Jelena S. Katanić Stanković, Daria Maria Monti, Enrica Giustino, Ljubinka G. Joksović, Sanja S. Krstić, Rudolf Bauer, Vladimir B. Mihailović

**Affiliations:** 1 Faculty of Science, Department of Chemistry, University of Kragujevac, Radoja Domanovića 12, 34000 Kragujevac, Serbia; nikola.sreckovic@pmf.kg.ac.rs (N.Z.S.); ljubinka.joksovic@pmf.kg.ac.rs (L.G.J.); 2 Institute for Information Technologies Kragujevac, Department of Science, University of Kragujevac, Liceja Kneževine Srbije 1A, 34000 Kragujevac, Serbia; jkatanic@kg.ac.rs; 3 Department of Chemical Sciences, Complesso Universitario Monte Sant’Angelo, University of Naples Federico II, via Cinthia 4, 80126 Naples, Italy; mdmonti@unina.it; 4 Department of Experimental Medicine, Section of Biotechnology, Medical Histology and Molecular Biology, University of Campania “Luigi Vanvitelli”, 80138 Naples, Italy; enrica.giustino@unicampania.it; 5 Institute for Pharmaceutical Sciences, Department of Pharmacognosy, University of Graz, Beethovenstrasse 8, 8010 Graz, Austria; sanja.krstic@uni-graz.at (S.S.K.); rudolf.bauer@uni-graz.at (R.B.)

**Keywords:** *Salvia verticillata* L. roots, extraction, response surface methodology, rosmarinic acid, antioxidant activity, tyrosinase inhibition, cytotoxicity, anti-inflammatory activity

## Abstract

Despite the well-established bioactivity of *Salvia verticillata* L. aerial parts, their roots represent an unexplored plant matrix. In this study, root extracts obtained by ethnopharmacological (EE), maceration (ME), and ultrasound-assisted (UE) extraction were examined. Ultrasound-assisted extraction was optimized using response surface methodology (RSM, CCD) to maximize total phenolic content (TPC) and rosmarinic acid (RA), and the optimized extract was compared with EE and ME. All extracts exhibited high levels of total phenolics, flavonoids, and phenolic acids, while RA was the dominant compound according to HPLC analysis (96.67–137.47 mg g^−1^ d.e.). UE yielded the highest TPC and exhibited the strongest antioxidant activity in most of the applied assays. All extracts exhibited concentration-dependent tyrosinase inhibitory activity, with UE and EE showing the strongest effects. Moderate antibacterial activity, particularly against Gram-positive strains, and limited antifungal effects were observed. Strong COX-1 inhibition (>70% at 50 μg/mL) confirmed the anti-inflammatory potential of the extracts, whereas slight COX-2 inhibition was observed. UE exhibited moderate cytotoxic activity against A431, SVT2, and HaCaT cells. These findings identify *S. verticillata* roots as a promising source of rosmarinic acid-rich extracts and point out the importance of the extraction procedure to tailor the biological activity of the extract.

## 1. Introduction

The medicinal value of *Salvia* species has been recognized since antiquity, making the genus one of the best-known groups of aromatic and medicinal plants. This long tradition is reflected in its name, derived from the Latin verb *salvare* (“to heal” or “to save”), highlighting the historical perception of these plants as valuable natural remedies. Throughout history, several *Salvia* species have been regarded as valuable medicinal herbs, and common sage (*S. officinalis*) was even referred to as herba sacra (“sacred herb”) in historical herbal literature. Today, *Salvia* species are widely used in traditional medicine, as well as in the food, cosmetic, and pharmaceutical industries owing to their rich phytochemical composition and diverse biological activities [1,2,3,4]. Among them, *Salvia verticillata* L., characterized by its distinctive purple inflorescences (lilac sage or purple rain), remains one of the less investigated members of the genus despite its traditional use and promising biological properties. Ethnobotanical records from Serbia indicate that infusions prepared from the aerial parts of *S. verticillata* have traditionally been used as expectorants, for oral cavity disinfection, and as poultices for wound healing [5]. Previous phytochemical studies have focused primarily on the aerial parts, where rosmarinic acid and salvianolic acid derivatives have been identified among the major phenolic constituents and are considered the main contributors to the reported antioxidant, antimicrobial, anti-inflammatory, and cytotoxic activities [6,7,8,9]. In contrast, the root of *S. verticillata* has received remarkably little scientific attention. Plant roots often exhibit metabolic profiles distinct from those of aerial parts and may accumulate bioactive compounds at concentrations different from those found in leaves, stems, or flowers. Nevertheless, information regarding the phytochemical composition of *S. verticillata* root, its extraction characteristics, and its biological activities remains scarce. This gap in current knowledge limits the evaluation of *S. verticillata* root as a potential source of rosmarinic acid-rich extracts and other bioactive compounds.

Among the phenolic compounds identified in *Salvia* species, rosmarinic acid has attracted particular attention because of its broad spectrum of biological activities. Besides its strong antioxidant capacity, numerous studies have demonstrated its antimicrobial, anti-inflammatory, antiviral, neuroprotective, cardioprotective, and anticancer activities, making it an attractive natural compound for pharmaceutical, nutraceutical, and cosmetic applications [7,10,11]. Owing to its pharmacological relevance and widespread occurrence in *Salvia* species, rosmarinic acid is frequently used as a marker compound in phytochemical characterization and extraction studies. Consequently, the development of efficient extraction procedures that maximize its recovery while preserving extract quality remains an important objective in natural product research.

The extraction technique is one of the key factors influencing the yield, chemical composition, and biological activity of plant extracts. Although conventional methods, such as maceration and traditional extraction procedures, remain widely used, ultrasound-assisted extraction has emerged as a rapid and environmentally friendly alternative that improves the recovery of bioactive compounds while reducing extraction time and solvent consumption [12]. Since the efficiency of ultrasound-assisted extraction depends on several interacting extraction parameters, optimization is essential. Response Surface Methodology (RSM) is widely applied for this purpose because it enables the simultaneous evaluation of multiple variables and their interactions while minimizing the number of required experiments [13,14]. The successful application of this approach to *Salvia* species further supports its suitability for maximizing the recovery of rosmarinic acid-rich extracts [15].

The aim of the present study was to optimize ultrasound-assisted extraction of *Salvia verticillata* L. root using Response Surface Methodology (RSM) and to evaluate the phytochemical composition and biological activities of the optimized ethanolic extract in comparison with extracts obtained by maceration (ME) and traditional ethnopharmacological extraction (EE). Total phenolic content and HPLC-determined rosmarinic acid concentration were used as response variables for extraction optimization. Phytochemical characterization included the determination of total phenolic, total flavonoid, and total phenolic acid contents, together with HPLC quantification of rosmarinic acid, while biological activities were evaluated using multiple antioxidant assays, a tyrosinase inhibition assay, antimicrobial testing, cyclooxygenase (COX-1 and COX-2) inhibition assays, and cytotoxicity on human cell lines. By integrating extraction optimization with phytochemical characterization and biological evaluation, this study contributes to a better understanding of *S. verticillata* root as a previously neglected plant organ with considerable potential for the development of value-added natural products. The findings highlight the potential of *S. verticillata* root as a promising source of rosmarinic acid-rich extracts for future pharmaceutical, nutraceutical, and cosmetic applications.

## 2. Results

### 2.1. Optimization of Ultrasound-Assisted Extraction

#### 2.1.1. Experimental Design and Model Fitting

Prior to the comparative analysis of the extraction techniques, ultrasound-assisted extraction (UE) was optimized using response surface methodology (RSM) to maximize total phenolic content (TPC) and rosmarinic acid (RA) concentration. UE was selected due to its shorter extraction time, lower solvent and energy consumption, and potential to improve extraction efficiency compared with conventional methods [4]. A central composite design (CCD) was employed to evaluate four independent factors at three coded levels (−1, 0, and +1): ethanol concentration (A; 20, 60, and 100%), solvent-to-solid ratio (B; 10, 25, and 40 mL g^−1^), extraction time (C; 10, 20, and 30 min), and extraction temperature (D; 25, 50, and 75 °C). These levels were selected based on preliminary experiments conducted prior to RSM optimization to provide suitable conditions for phenolic extraction and an appropriate range for evaluating the effects of each factor. Total phenolic content (TPC) and rosmarinic acid (RA) concentration were selected as the response variables. The experimental design and corresponding response values are presented in Table 1. The CCD comprised 30 experimental runs, including six center points, enabling the estimation of experimental error and evaluation of model adequacy.

Quadratic polynomial models were fitted to both responses based on the sequential model sum of squares analysis. A full quadratic model adequately described the TPC response, whereas a reduced quadratic model, obtained after elimination of non-significant terms, provided the best fit for the RA response. The regression statistics of the developed models are summarized in Table 2. Both models were statistically significant and showed non-significant lack-of-fit, indicating satisfactory agreement between the experimental and predicted responses. The TPC model exhibited higher goodness-of-fit and predictive ability than the RA model, as reflected by higher R^2^, adjusted R^2^, predicted R^2^, and Adeq. Precision values (Table 2). Nevertheless, the reduced quadratic model adequately described the experimental data for RA within the investigated experimental region.

Therefore, both models were considered suitable for subsequent response surface analysis and numerical optimization. Consequently, the models were used to visualize the response surfaces and determine the optimal extraction conditions.

#### 2.1.2. Effects of Extraction Variables on Total Phenolic Content

**Figure 1:**
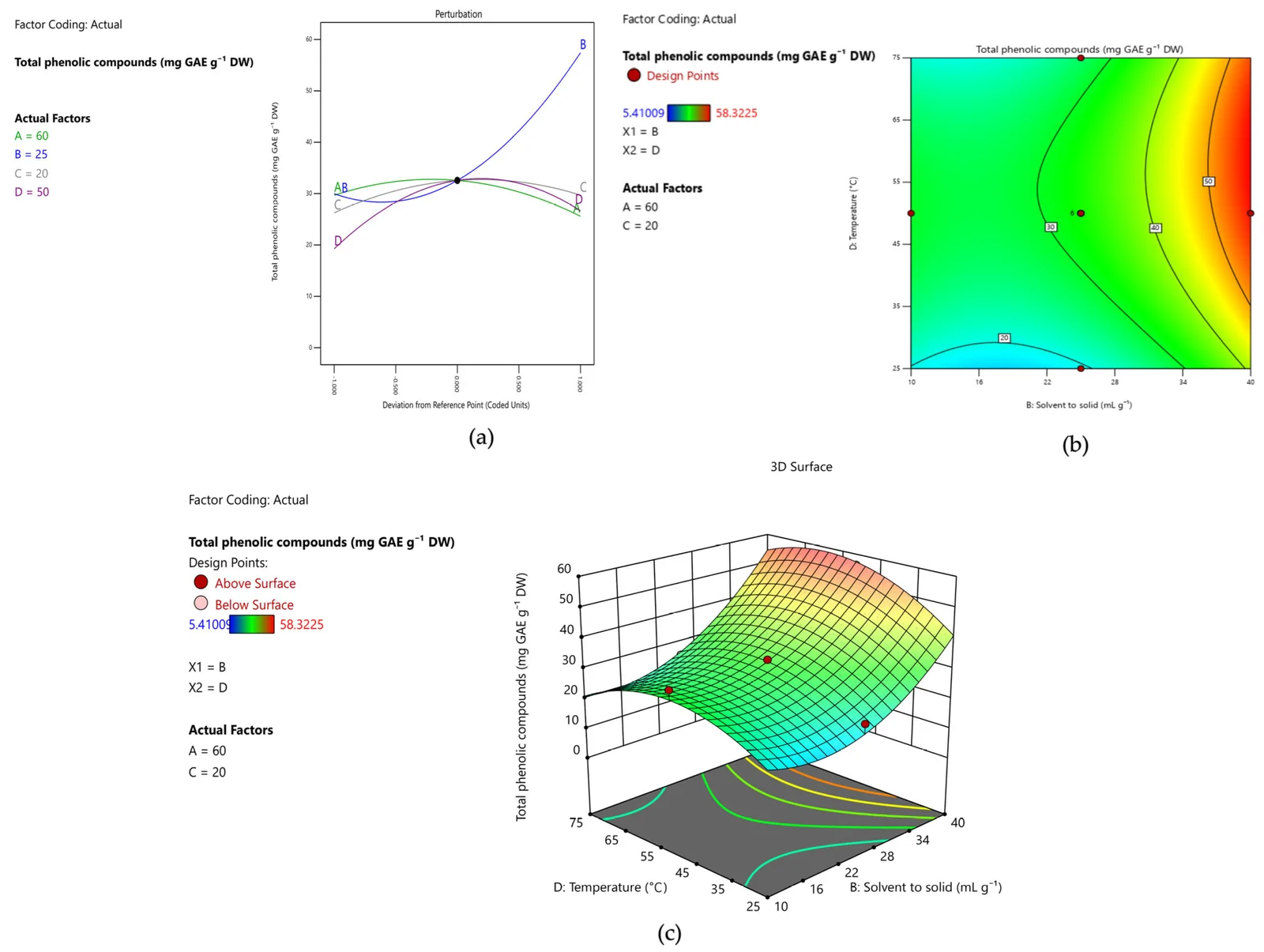
Response surface analysis of the effects of extraction variables on total phenolic content (TPC): (**a**) perturbation plot; (**b**) contour plot; and (**c**) three-dimensional (3D) response surface for the interaction between solvent-to-solid ratio and extraction temperature.

The effects of the extraction variables investigated on total phenolic content (TPC) are presented in Figure 1. The perturbation plot (Figure 1a) indicated that the solvent-to-solid ratio (B) exerted the greatest influence on TPC, followed by ethanol concentration (A), whereas extraction time (C) showed only a minor effect. Extraction temperature (D) also affected TPC, although to a lesser extent than the solvent-to-solid ratio.

The contour plot and three-dimensional response surface (Figure 1b,c) illustrated the interaction between solvent-to-solid ratio and extraction temperature. Increasing the solvent-to-solid ratio resulted in higher predicted TPC values across the investigated temperature range. Temperature exhibited a quadratic effect, with TPC increasing at higher temperatures, particularly when combined with elevated solvent-to-solid ratios. According to the developed model, the highest predicted TPC values were obtained under conditions combining elevated extraction temperatures with high solvent-to-solid ratios. These observations were consistent with the ANOVA results, which identified solvent-to-solid ratio, temperature, and their interaction (BD) as significant contributors to the developed model (*p* < 0.05).

#### 2.1.3. Effects of Extraction Variables on Rosmarinic Acid Concentration

**Figure 2:**
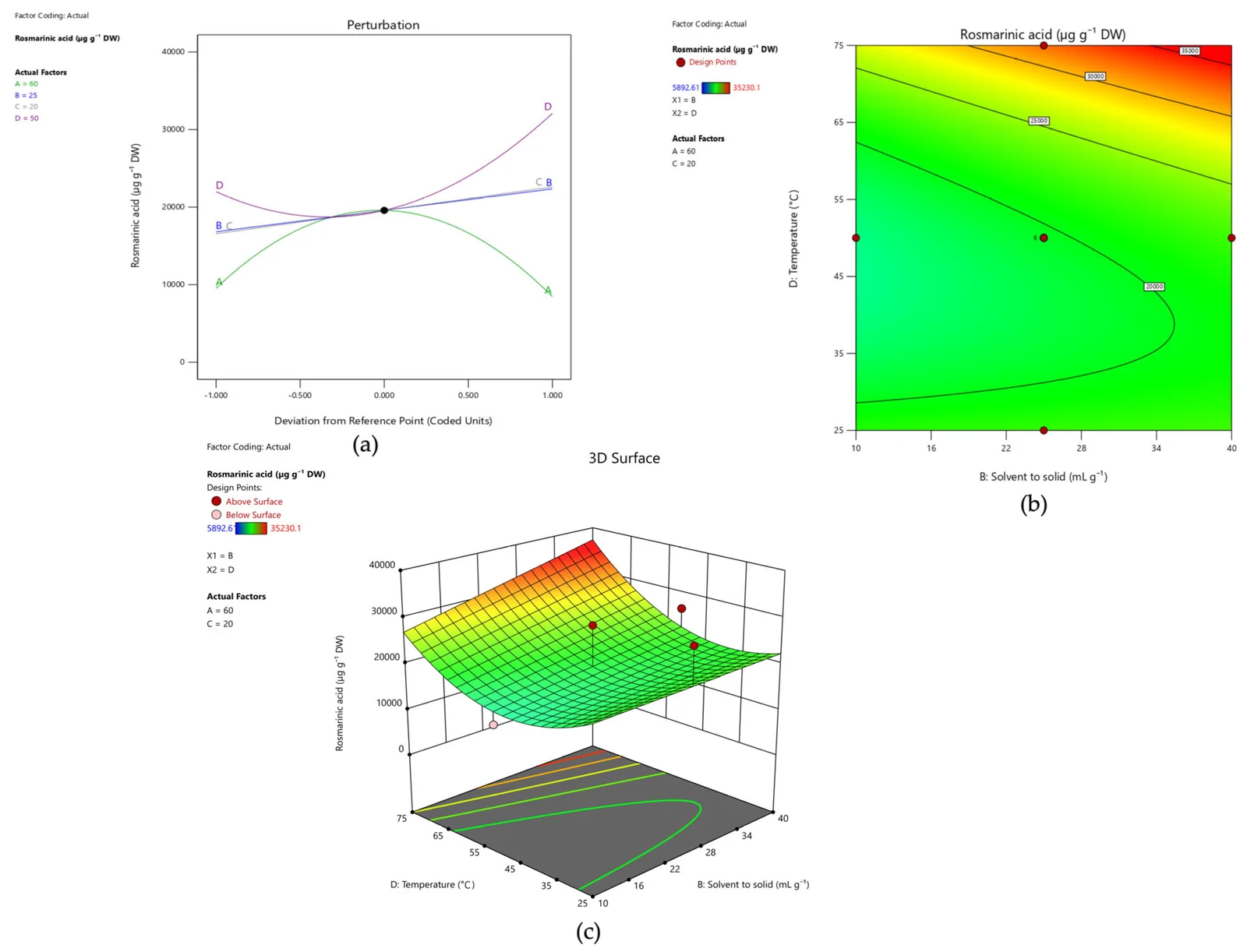
Response surface analysis of the effects of extraction variables on rosmarinic acid (RA): (**a**) perturbation plot; (**b**) contour plot; and (**c**) three-dimensional (3D) response surface for the interaction between solvent-to-solid ratio and extraction temperature.

The effects of the investigated extraction variables on rosmarinic acid (RA) concentration are presented in Figure 2. The perturbation plot (Figure 2a) showed that extraction temperature (D) exerted the greatest influence on RA concentration, followed by ethanol concentration (A), whereas solvent-to-solid ratio (B) had a moderate positive effect and extraction time (C) had only a minor effect on RA concentration.

The contour and response surface plots (Figure 2b,c) further demonstrated significant interaction between solvent-to-solid ratio and extraction temperature (*p* < 0.05). Increasing the solvent-to-solid ratio resulted in higher predicted RA concentrations, particularly at elevated extraction temperatures. Conversely, lower RA concentrations were predicted at low solvent-to-solid ratios across the investigated temperature range. According to the developed model, RA concentration was maximized under conditions combining high extraction temperatures with high solvent-to-solid ratios. These observations were in agreement with the reduced quadratic model, in which temperature, solvent-to-solid ratio, and their interaction (BD) significantly affected RA concentration (*p* < 0.05).

#### 2.1.4. Numerical Optimization

**Figure 3:**
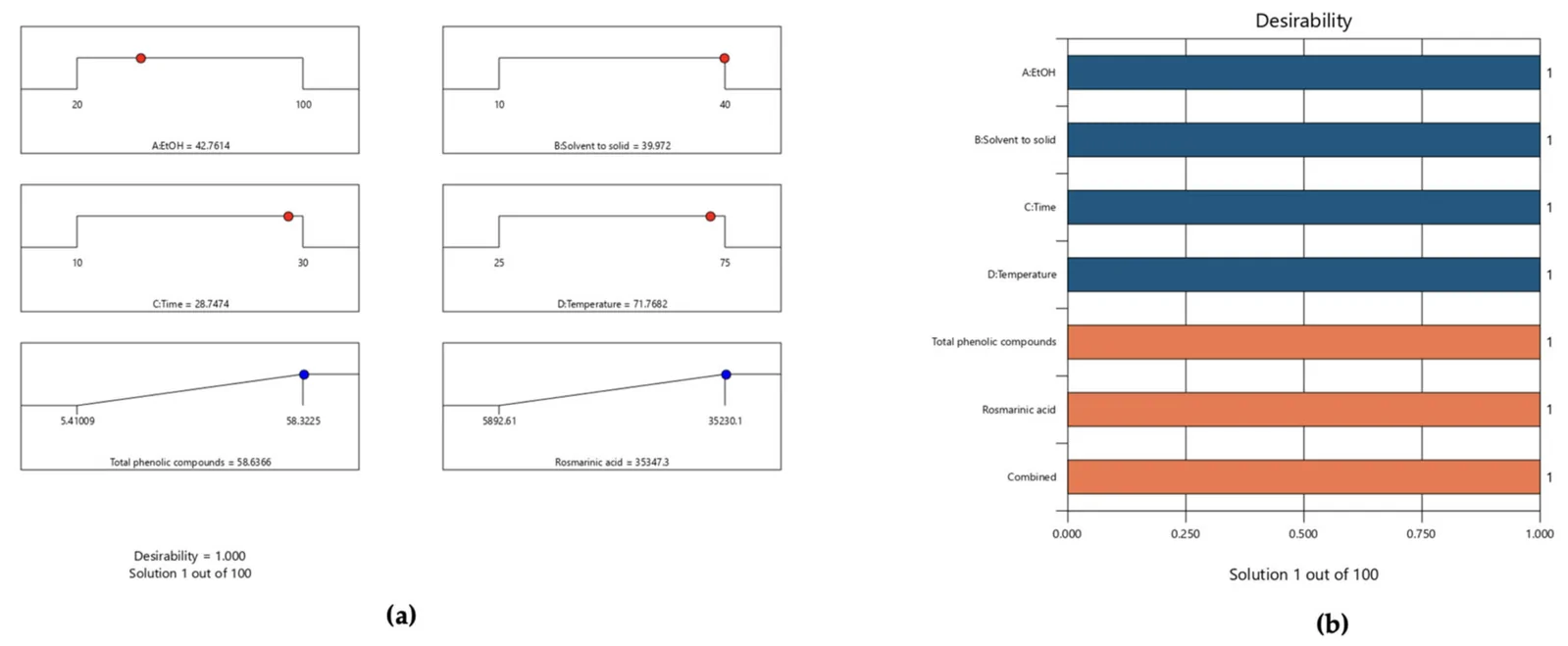
Numerical optimization of ultrasound-assisted extraction based on the desirability function: (**a**) Optimal extraction conditions and predicted values of total phenolic content (TPC) and rosmarinic acid (RA); (**b**) Overall desirability plot for the optimized extraction conditions; blue bars represent process factors, while red bars represent response variables and combined desirability.

Numerical optimization was performed using the desirability function approach in order to simultaneously maximize total phenolic content (TPC) and rosmarinic acid (RA) concentration while keeping all extraction variables within the investigated experimental ranges. The optimization criteria used for each factor and response are summarized in Table 3. The optimal extraction conditions predicted by the desirability function were an ethanol concentration of 42.76%, solvent-to-solid ratio of 39.997 mL g^−1^, extraction time of 28.75 min, and extraction temperature of 71.77 °C, yielding an overall desirability of 1.000 (Figure 3). At these conditions, the predicted TPC and RA values were 58.64 mg GAE g^−1^ DW and 35,358.2 μg g^−1^ DW, respectively, representing the simultaneous maximization of both response variables within the investigated design space.

**Figure 4:**
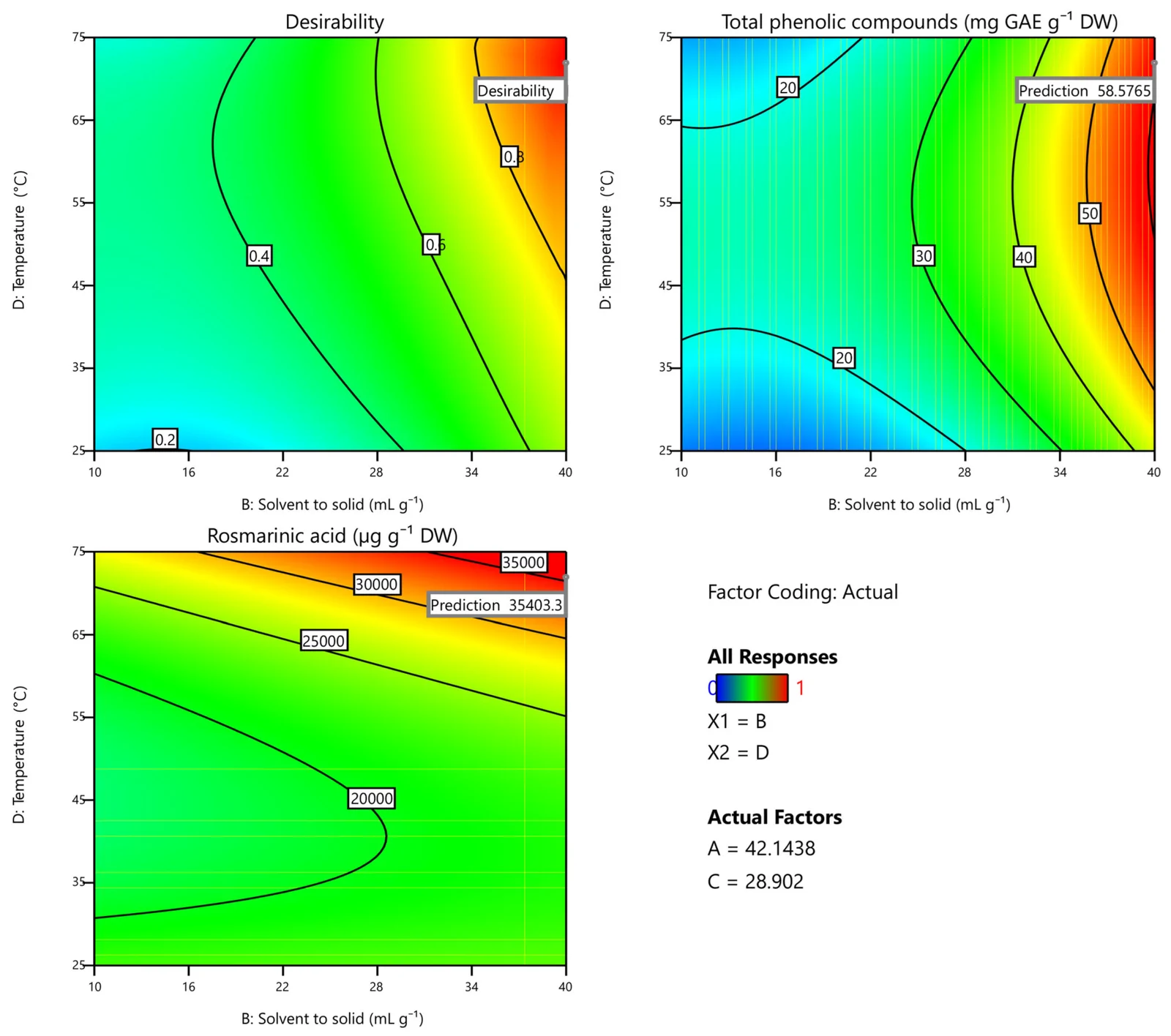
Combined desirability contour plots illustrating the optimal experimental region for simultaneous maximization of total phenolic content (TPC) and rosmarinic acid (RA).

The combined desirability contour plots (Figure 4) further confirmed that the optimal region corresponded to a high solvent-to-solid ratio and elevated extraction temperature, where both response variables simultaneously reached their highest predicted values.

These optimized extraction conditions were subsequently used for the preparation of the UE extract evaluated in comparative phytochemical and biological analyses.

### 2.2. Phenolic Composition of S. verticillata Root Extracts

**Figure 5:**
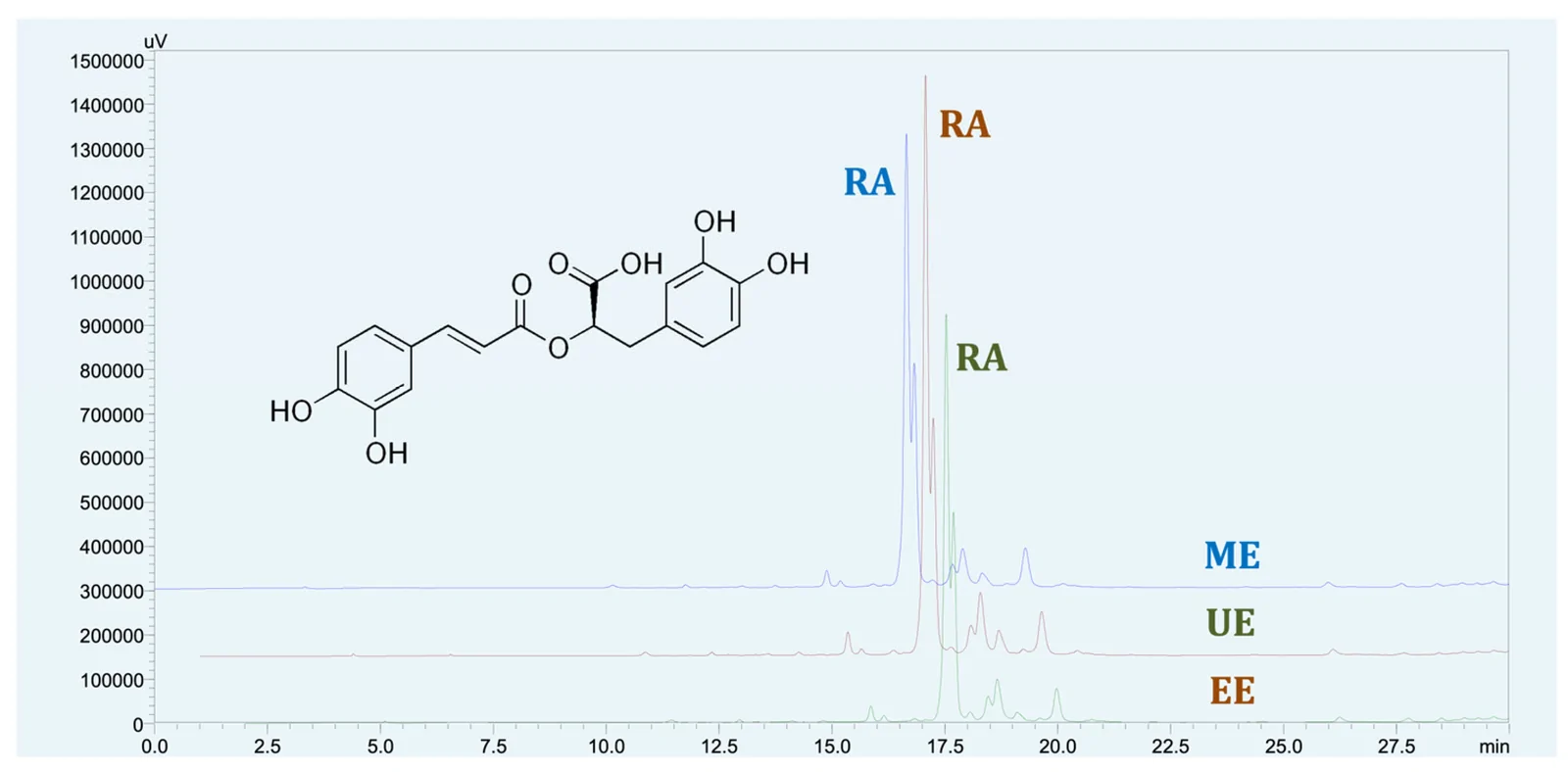
Representative HPLC-PDA chromatograms showing the rosmarinic acid (RA) peak in ethnopharmacological (EE), maceration (ME), and optimized ultrasound-assisted (UE) extracts of *S. verticillata* roots.

The phenolic composition of the *S. verticillata* root extracts prepared by ethnopharmacological extraction (EE), maceration (ME), and optimized ultrasound-assisted extraction (UE) was evaluated by spectrophotometric assays and HPLC-PDA analysis. The quantitative results are summarized in Table 4, while representative HPLC chromatograms are presented in Figure 5.

The highest TPC was observed in UE (313.9 mg GAE g^−1^ dry extract), although it did not differ significantly from EE (304.2 mg GAE g^−1^ dry extract) (*p* > 0.05). Both extracts contained significantly higher amounts of total phenolics than ME (*p* < 0.05). EE showed the highest TFC (47.2 mg RUE g^−1^ dry extract) and TPA (22.4 mg CAE g^−1^ dry extract), while UE exhibited similar TPA values (*p* > 0.05). In contrast, ME had significantly lower contents of all spectrophotometrically determined phenolic groups (*p* < 0.05). Rosmarinic acid was the predominant phenolic compound in all extracts. Its concentration was highest in UE (137.47 mg g^−1^ dry extract), followed by EE (129.26 mg g^−1^ dry extract) and ME (96.67 mg g^−1^ dry extract), with significant differences among all three extracts (*p* < 0.05).

The representative chromatograms confirmed the predominance of rosmarinic acid in all extracts, with the highest peak intensity observed for UE, followed by EE and ME, in agreement with the quantitative HPLC results.

### 2.3. Antioxidant Activity of S. verticillata Root Extracts

The antioxidant activity of *S. verticillata* root extracts was evaluated using total antioxidant capacity (TAC), reducing power (RP), DPPH, and ABTS assays. The results are summarized in Table 5.

The antioxidant activity differed among the extracts depending on the assay employed. EE exhibited the highest total antioxidant capacity (1368.9 mg AA g^−1^ dry extract), followed by UE and ME, with significant differences among all extracts (*p* < 0.05). In contrast, UE showed the greatest reducing power (957.3 mg Trolox g^−1^ dry extract), significantly exceeding both EE and ME (*p* < 0.05).

The DPPH assay further confirmed the superior antioxidant potential of UE, which exhibited the lowest IC_50_ value (10.2 μg mL^−1^), followed by EE (17.4 μg mL^−1^) and ME (35.6 μg mL^−1^), with all values differing significantly (*p* < 0.05). Notably, UE displayed significantly stronger DPPH radical scavenging activity than ascorbic acid (IC_50_ = 20.6 μg mL^−1^) (*p* < 0.05), whereas EE was also more active than the reference compound and ME was less active. In the ABTS assay, EE and UE showed comparable radical scavenging activities (*p* > 0.05), and both were significantly more active than ME (*p* < 0.05). Overall, the optimized ultrasound-assisted extract demonstrated the strongest antioxidant performance, particularly in the reducing power and DPPH assays.

### 2.4. Tyrosinase Inhibitory Activity of S. verticillata Root Extracts

**Figure 6:**
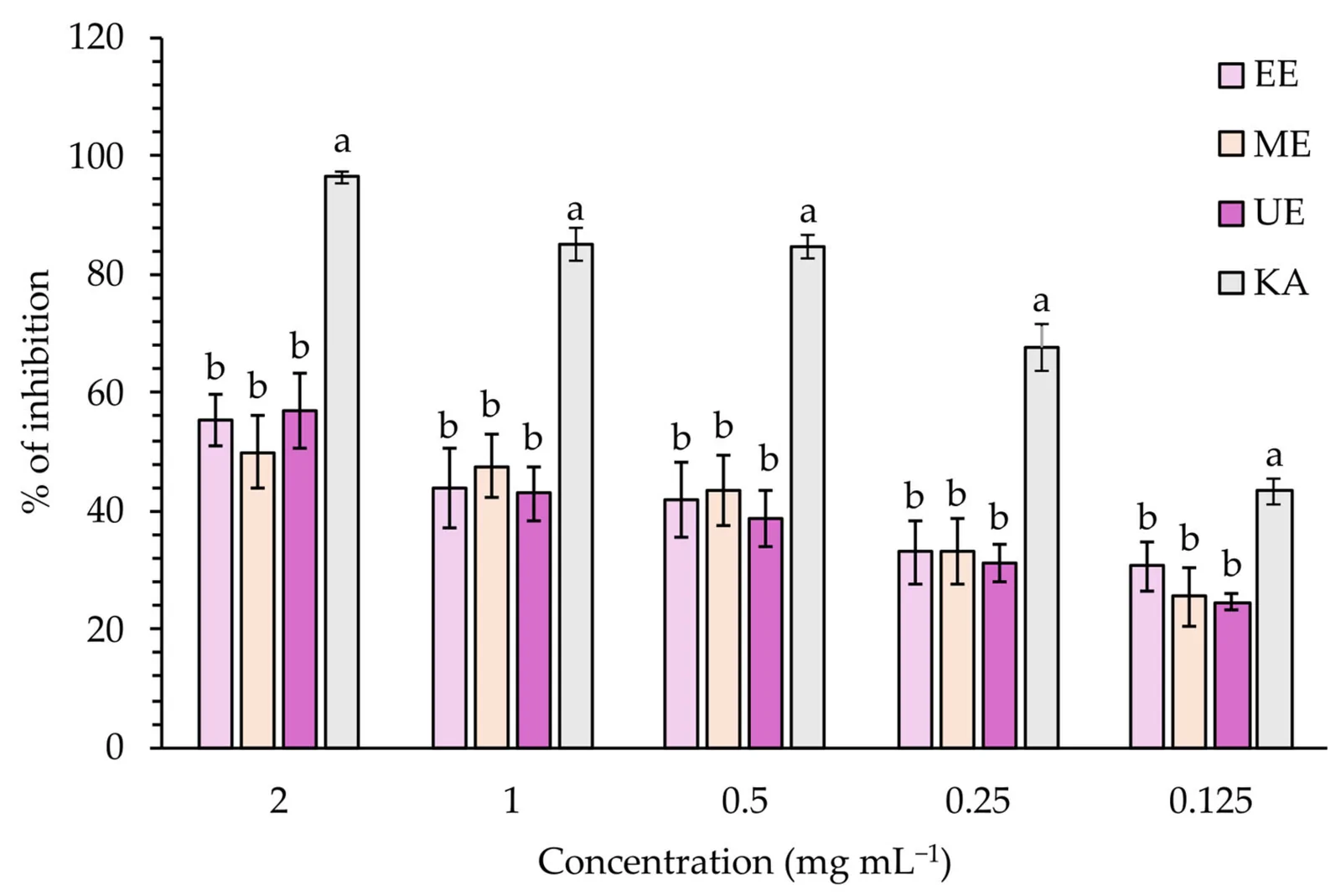
Tyrosinase inhibitory activity of *S. verticillata* root extracts at different concentrations (EE—ethnopharmacological extraction; ME—maceration; UE—optimized ultrasound-assisted extraction; KA—kojic acid). Different superscript letters at the same tested concentration indicate statistically significant differences (*p* < 0.05).

Tyrosinase inhibitory activity of *S. verticillata* root extracts was evaluated at concentrations ranging from 0.125 to 2 mg mL^−1^ (Figure 6).

All extracts inhibited tyrosinase in a concentration-dependent manner. At 2 mg mL^−1^, UE exhibited the highest inhibitory activity (56.95%), closely followed by EE (55.42%) and ME (50.01%), although no significant differences were observed among the extracts at any of the tested concentrations (*p* > 0.05). In contrast, kojic acid (KA), used as the positive control, showed significantly stronger inhibition than all extracts (*p* < 0.05), reaching 96.56% at 2 mg mL^−1^ and remaining above 40% even at the lowest tested concentration.

This trend was also reflected in the IC_50_ values, with UE showing the lowest value (1.37 mg mL^−1^), followed by EE (1.40 mg mL^−1^) and ME (1.50 mg mL^−1^). However, these differences were small and consistent with the comparable inhibitory activities observed among the extracts (*p* > 0.05). As expected, kojic acid exhibited the lowest (*p* < 0.05) IC_50_ overall (0.12 mg mL^−1^).

### 2.5. Antimicrobial Activity of S. verticillata Root Extracts

The antimicrobial activity of *S. verticillata* root extracts was evaluated against eleven bacterial and nine fungal strains. The minimum inhibitory concentration (MIC) values are summarized in Table 6.

Among the tested microorganisms, the extracts exhibited greater activity against bacteria than fungi. Among the bacterial strains, *B. subtilis* was the most susceptible, with MIC values below 0.156 mg mL^−1^ for all three extracts. A pronounced antibacterial effect was also observed against *S. aureus*, particularly for EE (MIC = 0.156 mg mL^−1^), while ME and UE showed identical MIC values of 0.3125 mg mL^−1^. Compared with ME and UE, EE exhibited lower MIC values against *S. typhimurium*, *B. cereus*, and *K. pneumoniae*. For the remaining bacterial strains, MIC values ranged from 10 to 20 mg mL^−1^.

Overall, the extracts showed limited antifungal activity. UE showed the strongest activity against *C. albicans* (MIC = 10 mg mL^−1^), whereas EE and ME inhibited this strain at 20 mg mL^−1^. All three extracts produced MIC values of 20 mg mL^−1^ against the remaining fungal strains, while *A. alternata* was not inhibited at the highest tested concentration (MIC > 20 mg mL^−1^). Taken together, EE demonstrated the strongest antibacterial activity, whereas UE showed slightly higher antifungal activity against *C. albicans*.

### 2.6. Anti-Inflammatory Activity of S. verticillata Root Extracts

The results presented in Table 7 demonstrate that all investigated *Salvia* extracts exhibited pronounced COX-1 inhibitory activity, with inhibition values of 71.8% for EE, 80.2% for ME, and 79.8% for UE. Among the tested extracts at a concentration of 50 μg mL^−1^, ME and UE showed significantly higher COX-1 inhibitory activity than EE (*p* < 0.05), while no significant difference was observed between ME and UE (*p* > 0.05). Rosmarinic acid (10 μM) also exhibited considerable COX-1 inhibition (73.8%) and did not differ significantly from any of the tested extracts (*p* > 0.05). Indomethacin (1.25 μM) showed the highest COX-1 inhibition (85.1%); however, its activity did not differ significantly from that of ME and UE (*p* > 0.05).

In contrast, the extracts exhibited lower inhibitory activity against COX-2, with inhibition values of 56.5% for EE, 42.8% for ME, and 42.9% for UE. EE showed significantly higher COX-2 inhibitory activity than ME and UE (*p* < 0.05), while no significant difference was observed between ME and UE (*p* > 0.05). Rosmarinic acid (10 μM) showed the lowest COX-2 inhibitory activity (19.4%), which was significantly lower than that of all three extracts (*p* < 0.05). Celecoxib (8.8 μM) exhibited the highest COX-2 inhibition (83.6%), significantly exceeding that of all tested extracts and rosmarinic acid (*p* < 0.05). Overall, the extracts investigated exhibited greater inhibitory activity toward COX-1 than toward COX-2 under the applied experimental conditions. Notably, ME and UE showed COX-1 inhibitory activities statistically comparable to that of the reference anti-inflammatory drug indomethacin (*p* > 0.05), whereas substantially lower inhibitory activities were observed against COX-2.

This difference may be explained by the assumption that the bioactive constituents responsible for the anti-inflammatory activity possess a higher binding affinity for the active site of COX-1 than for COX-2, resulting in more effective enzyme inhibition. This hypothesis could be further supported and validated through molecular docking analysis, which would provide insights into the binding interactions and affinities of the identified bioactive compounds toward both cyclooxygenase isoenzymes.

### 2.7. Cytotoxic Activity of S. verticillata Root Extracts

Based on the results of the phytochemical characterization and biological activity assays, the ethnopharmacological (EE) and optimized ultrasound-assisted (UE) extracts were selected for subsequent cytotoxicity evaluation.

**Figure 7:**
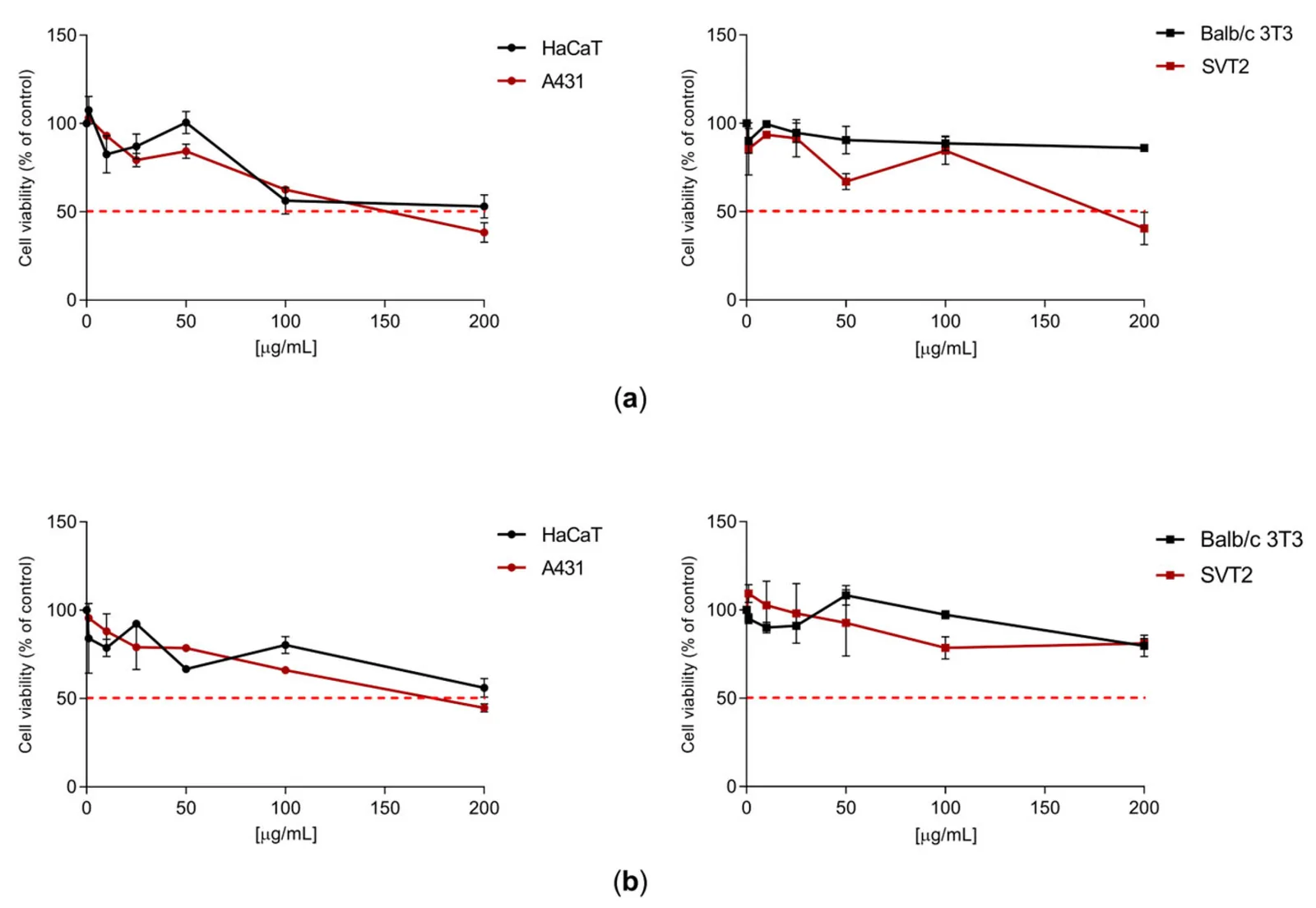
Dose–response curves of immortalized and cancer cell lines after 72 h incubation with increasing concentrations of UE (**a**) and EE (**b**) extracts on HaCaT (black circles), A431 (red circles), Balb/c-3T3 (black squares) and SVT2 (red squares) cells. Cell viability was assessed by the MTT assay and expressed as the percentage of viable cells relative to untreated control cells. Data are shown as means ± SD of three independent experiments.

The cytotoxic effects of these extracts were assessed by the MTT assay using two normal cell lines (human HaCaT keratinocytes and murine Balb/c-3T3 fibroblasts) and two tumor cell lines (human A431 epidermoid carcinoma cells and murine SVT2 transformed fibroblasts). The corresponding dose–response curves are shown in Figure 7, and the calculated IC_50_ values are presented in Table 8.

As shown in Figure 7a, the UE extract reduced cell viability in A431, SVT2, and HaCaT cells, with IC_50_ values (Table 8) of 155, 191 and 160 µg mL^−1^, respectively, indicating moderate cytotoxic activity within the tested concentration range. In contrast, no IC_50_ value was reached for Balb/c-3T3 cells. Notably, the comparable IC_50_ values obtained for A431 and HaCaT cells indicate that UE did not exhibit preferential cytotoxicity toward the A431 cancer cell line. The EE extract (Figure 7b) showed moderate cytotoxic activity only toward A431 cells (IC_50_ = 183 µg mL^−1^), whereas no IC_50_ value was reached for HaCaT, Balb/c-3T3, or SVT2 cells within the tested concentration range.

## 3. Discussion

Response surface methodology proved to be an effective approach for optimizing the extraction of phenolic compounds from *S. verticillata* roots. All the analyses indicated the optimized UE protocol as the one able to yield the highest total phenolic content and RA concentration among the investigated extraction methods. The developed quadratic models adequately described the effects of ethanol concentration, solvent-to-solid ratio, extraction time, and extraction temperature on total phenolic content (TPC) and rosmarinic acid (RA) recovery, enabling simultaneous optimization of both responses using the desirability function approach. Similar studies have demonstrated that RSM is a reliable statistical tool for optimizing extraction processes because it enables simultaneous evaluation of multiple variables and their interactions while considerably reducing the number of experimental trials required to establish optimal extraction conditions. This has also been confirmed in recent optimization studies on *Salvia* species, including *S. deserta* and *S. fruticosa*, where RSM successfully maximized the recovery of phenolic compounds under optimized extraction conditions [12,13,15,16].

Among the investigated extraction variables, solvent-to-solid ratio exerted the greatest influence on TPC, whereas extraction temperature had the strongest effect on RA recovery. This agrees with the high rosmarinic acid concentration obtained in the optimized extract and emphasizes the importance of temperature for efficient RA recovery under the investigated conditions. The positive influence of increasing the solvent-to-solid ratio agrees with the mass-transfer mechanism governing solid–liquid extraction, where a larger solvent volume enhances the concentration gradient between the plant matrix and the extraction medium, thereby facilitating diffusion of soluble phenolic compounds [14,16]. Similarly, increasing extraction temperature improves solvent penetration and mass transfer, although excessive temperatures may also promote partial degradation of thermolabile phenolic constituents [15,16,17]. Extraction time exerted only a minor influence on both TPC and RA, suggesting that most extractable phenolic compounds were released during the initial stages of ultrasound-assisted extraction, while prolonged sonication provided little additional benefit under the investigated conditions. These observations are consistent with previous optimization studies on *Salvia* species, where extraction conditions, including ethanol concentration, extraction time, temperature, ultrasonic amplitude, and solvent-related parameters, were identified as important determinants of phenolic recovery and antioxidant activity [15,16,18,19]. The optimum ethanol concentration of approximately 42.8% obtained in the present study is also in agreement with previous reports demonstrating that hydroethanolic mixtures containing approximately 40–60% ethanol provide favorable polarity for simultaneous extraction of structurally diverse phenolic compounds [14,17,18]. Water promotes swelling of plant tissues and facilitates solvent penetration, whereas ethanol enhances the solubility of moderately polar phenolic constituents. Consequently, intermediate ethanol concentrations frequently provide higher extraction efficiency than either pure water or absolute ethanol [14,15].

Besides improving extraction efficiency, the optimized extraction conditions enabled recovery of the highest amounts of rosmarinic acid and total phenolics within a relatively short extraction time [12,17]. In the present study, numerical optimization identified extraction conditions that achieved the highest predicted TPC and RA values within the investigated experimental domain in less than 30 min. Experimental validation confirmed these predictions, with the optimized protocol producing the extract with the highest total phenolic content and rosmarinic acid concentration among the investigated extraction methods. Comparable agreement between predicted and experimental values has been reported in RSM optimization studies on *S. deserta* and *S. fruticosa*, further supporting the reliability of RSM for predicting extraction performance in *Salvia* species [15,16]. Moreover, the desirability value of 1.000 indicated that the selected extraction conditions simultaneously maximized both responses without compromising either TPC or RA recovery, confirming the robustness of the optimization strategy. The satisfactory predictive performance of the developed models further supports the suitability of response surface methodology for establishing efficient extraction conditions. These findings support the application of optimized ultrasound-assisted extraction for subsequent phytochemical and biological characterization of *S. verticillata* root extracts.

Comparative phytochemical analysis demonstrated that the extraction method influenced both the quantity and composition of phenolic constituents in *S. verticillata* root extracts. The optimized UE extract contained significantly higher rosmarinic acid concentration than EE and ME (*p* < 0.05), while its total phenolic content was comparable to that of EE (*p* > 0.05) and significantly higher than that of ME (*p* < 0.05). In contrast, EE exhibited significantly higher total flavonoid content than both UE and ME (*p* < 0.05), whereas its total phenolic acid content was comparable to that of UE (*p* > 0.05) but significantly higher than that of ME (*p* < 0.05), indicating distinct extraction selectivity toward different phenolic subclasses. Although previous studies have focused primarily on the aerial parts of *S. verticillata*, they likewise identified rosmarinic acid as one of the predominant phenolic constituents [7,8], supporting the present findings obtained for the root extracts. The superior performance of the UE extract is likely related to acoustic cavitation, which enhances solvent penetration and facilitates the release of intracellular metabolites [12,17]. Prolonged extraction may favor the recovery of certain phenolic subclasses, particularly flavonoids, whereas ultrasound-assisted extraction proved more efficient for recovering rosmarinic acid. Comparable observations have also been reported for *S. deserta* and *S. officinalis*, where extraction efficiency depended on both extraction conditions and the physicochemical properties of individual metabolites [16,18]. Likewise, Brudiu et al. [19] demonstrated that optimized ultrasound-assisted extraction of *S. transsylvanica* yielded phenolic-rich extracts with high antioxidant potential.

Rosmarinic acid is considered one of the predominant phenolic compounds in *Salvia* species and is frequently used as a phytochemical marker because of its well-documented biological activities [7,10,20]. Katanić Stanković et al. [8] identified rosmarinic acid as the major phenolic constituent of methanolic extracts of *S. verticillata* aerial parts, reporting approximately 235 mg g^−1^ extract. Although direct comparison should be interpreted cautiously because different plant organs, extraction procedures, and analytical conditions were used, the optimized UE root extract obtained in the present study contained 137.47 mg g^−1^ extract, indicating that the roots of *S. verticillata* are also a rich source of rosmarinic acid. Lower concentrations were reported for methanolic extracts of both the aerial parts (8.42 mg g^−1^ dry extract) and roots (11.07 mg g^−1^ dry extract) of *S. pratensis* [21]. *Salvia officinalis* has also been reported to contain 39.3 mg g^−1^ dried plant of rosmarinic acid [22]. Despite containing the highest concentration of rosmarinic acid, the antioxidant activity of the UE extract cannot be attributed solely to this compound. Although the total phenolic content of the UE extract was comparable to that of the EE extract (*p* > 0.05), UE exhibited the highest reducing power and the strongest DPPH radical scavenging activity (*p* < 0.05), suggesting that its antioxidant potential resulted from the combined contribution of numerous phenolic constituents acting additively or synergistically [20,23].

The differences observed among the applied antioxidant assays further support this interpretation. Because the antioxidant assays are based on different reaction mechanisms, differences among them were expected and reflect the complexity of the extract composition rather than the concentration of a single phenolic constituent [24,25,26]. These observations are consistent with the significantly higher reducing power and stronger DPPH radical scavenging activity of the UE extract compared with EE (*p* < 0.05). In contrast, EE exhibited significantly higher TAC than UE (*p* < 0.05), whereas their ABTS radical scavenging activities were comparable (*p* > 0.05). Similarly, a previous study on the aerial parts of *S. verticillata* reported a high rosmarinic acid content together with pronounced antioxidant activity, further supporting the relationship between phenolic composition and antioxidant potential observed in the present study [8]. The present findings indicate that optimization of ultrasound-assisted extraction improved not only the recovery of total phenolics but also the extraction efficiency of rosmarinic acid, emphasizing the importance of combining optimized extraction procedures with comprehensive phytochemical characterization and complementary antioxidant assays. This approach provides a more reliable assessment of the antioxidant potential of plant extracts than reliance on a single phenolic marker.

Tyrosinase (EC 1.14.18.1) is a copper-containing oxidase present in microorganisms, plants, and animals that plays a central role in melanin biosynthesis. It catalyzes the conversion of L-tyrosine to L-DOPA and the subsequent oxidation of L-DOPA to dopaquinone, the initial steps of the melanogenic pathway. In addition to its physiological function in pigmentation, tyrosinase is responsible for enzymatic browning in fruits and vegetables, which adversely affects their color and overall quality. Consequently, the development of effective tyrosinase inhibitors has attracted considerable interest for applications in the cosmetic, pharmaceutical, and food industries, with kojic acid, hydroquinone, arbutin, and azelaic acid being among the best-known examples [27]. The search for naturally occurring tyrosinase inhibitors has therefore become an active area of research. In the present study, all *S. verticillata* root extracts inhibited tyrosinase in a concentration-dependent manner. At the highest tested concentration (2 mg mL^−1^), UE exhibited the strongest inhibitory activity (56.95%), closely followed by EE (55.42%), whereas ME showed somewhat lower inhibition (50.01%), but with no statistical differences (*p* > 0.05). This trend was also reflected in the IC_50_ values, with UE showing the lowest value (1.37 mg mL^−1^), followed by EE (1.40 mg mL^−1^) and ME (1.50 mg mL^−1^). As expected, kojic acid, used as the positive control, exhibited substantially higher (*p* < 0.05) inhibitory activity (96.56%) and a considerably lower IC_50_ value (0.12 mg mL^−1^). The slightly higher activity of UE may be related to differences in the phytochemical composition of the extracts, as revealed by the phytochemical analyses. The IC_50_ values of the three extracts differed only slightly (1.37–1.50 mg mL^−1^), indicating that the extraction method had a limited influence on the overall tyrosinase inhibitory potency. However, the relatively modest differences in tyrosinase inhibition despite more pronounced differences in phytochemical composition suggest that the observed activity is more likely determined by the combined action of multiple constituents than by the abundance of a single compound.

Although information on the tyrosinase inhibitory activity of *Salvia* species remains limited, several species have demonstrated the ability to inhibit this enzyme. Methanolic leaf extracts of *S. officinalis* exhibited significantly higher tyrosinase inhibitory activity than chloroform extracts, which was associated with their higher phenolic content. Rosmarinic acid was identified as the predominant phenolic compound in the *S. officinalis* methanolic extract, reaching 46.02 mg g^−1^ extract [28]. Likewise, methanolic extracts obtained from five Turkish *Salvia* taxa showed measurable tyrosinase inhibitory activity, although substantially lower than that of kojic acid. At a concentration of 50 µg/mL, *S. aytachii* exhibited the highest inhibition (16.29%), whereas kojic acid inhibited tyrosinase activity by 88.43% under the same experimental conditions [29]. Although direct comparison with the present study should be interpreted cautiously because of differences in plant species, plant organs, extraction procedures, extract concentrations, and assay protocols, these studies demonstrate that tyrosinase inhibition is a common characteristic of phenolic-rich *Salvia* extracts. Rosmarinic acid has been identified as one of the compounds capable of directly inhibiting tyrosinase. Kang et al. reported that rosmarinic acid isolated from *S. miltiorrhiza* inhibited mushroom tyrosinase with an IC_50_ value of 16.8 μM, comparable to that of kojic acid (22.4 μM), while kinetic analysis showed that it acts as a competitive inhibitor [30]. However, subsequent spectrum-effect relationship analysis and molecular docking demonstrated that tyrosinase inhibition in *S. miltiorrhiza* is associated with several phenolic constituents rather than a single compound, with protocatechuic aldehyde, hydroxysafflor yellow A, and tanshinone IIA also contributing to the observed activity [31]. Therefore, although rosmarinic acid may contribute to the tyrosinase inhibitory activity of *S. verticillata* extracts, the present results suggest that the observed activity is more likely determined by the combined action of multiple phytochemicals. Taken together, these findings indicate that *S. verticillata* root represents a promising natural source of tyrosinase inhibitors and warrants further investigation for cosmetic applications aimed at managing skin hyperpigmentation.

The antimicrobial activity of *S. verticillata* root extracts (Table 6) was generally more pronounced against Gram-positive bacteria than against Gram-negative bacteria, whereas Gram-negative bacteria and the majority of fungal strains exhibited considerably lower susceptibility. Among the tested microorganisms, *Bacillus subtilis* and *Staphylococcus aureus* were the most susceptible bacterial species, while among the investigated fungi, only *Candida albicans* showed moderate susceptibility. Although the antibacterial activities of the ethnopharmacological extract (EE) and the optimized ultrasound-assisted extract (UE) were generally comparable, the EE extract showed greater activity against several bacterial strains, including *Klebsiella pneumoniae*, *Bacillus cereus*, *Staphylococcus aureus*, and *Salmonella Typhimurium*, despite the UE extract containing the highest total phenolic and rosmarinic acid contents. This difference may be attributed to the distinct phenolic profiles of the extracts investigated. The EE extract contained higher amounts of total flavonoids and total phenolic acids, whereas the UE extract was characterized by the highest total phenolic and rosmarinic acid contents. Collectively, these observations suggest that antibacterial activity is influenced by the overall phytochemical composition and possible synergistic interactions among phenolic constituents rather than by the abundance of a single compound or the total phenolic content.

Srećković et al. [21] reported pronounced antibacterial activity of methanolic root extracts of *S. pratensis*, particularly against Gram-positive bacteria and *Salmonella typhimurium* (MIC < 0.156 mg/mL), whereas antifungal activity was considerably weaker, with MIC values ranging from 2.5 to 20 mg/mL. Similarly, hydromethanolic root extracts of *S. cadmica* exhibited strong antibacterial activity against Gram-positive bacteria, especially species belonging to the genera *Staphylococcus* (MIC 0.156–0.625 mg/mL) and *Bacillus* (MIC 0.156 mg/mL), which is consistent with the higher susceptibility of *S. aureus* and *B. subtilis* observed in the present study [32]. These studies suggest that root-derived extracts within the genus *Salvia* generally possess greater antibacterial than antifungal potential. Katanić Stanković et al. [8] investigated methanolic aerial-part extracts and observed the highest antibacterial activity against *Bacillus cereus*, whereas Gram-negative bacteria and *Candida albicans* were considerably less susceptible. Although a different plant organ was investigated, the susceptibility pattern was similar to that observed in the present study, with Gram-positive bacteria being more sensitive than Gram-negative bacteria and fungi. Comparable findings were reported for root extracts of *S. verticillata*, where chloroform extracts exhibited greater activity against Gram-positive bacteria than against fungi [33]. Collectively, these reports highlight that extraction solvent, plant organ, and phytochemical composition markedly influence the antimicrobial activity of *S. verticillata* extracts. The observed antimicrobial activity is likely associated with the combined action of phenolic acids, flavonoids, and other bioactive constituents, such as diterpenes, which have been reported to disrupt microbial cell membranes, increase membrane permeability, interfere with essential enzymatic processes, and ultimately inhibit microbial growth [7]. The greater susceptibility of Gram-positive bacteria can be explained by the absence of an outer lipopolysaccharide membrane, whereas the outer membrane of Gram-negative bacteria acts as an effective permeability barrier that restricts the penetration of many bioactive phytochemicals [34]. The present study indicates that *S. verticillata* root extracts possess selective antimicrobial activity directed primarily toward Gram-positive bacteria, with the stronger antibacterial activity of the EE extract suggesting that antimicrobial efficacy is more likely governed by the overall phenolic profile and possible synergistic interactions among individual constituents than solely by the total concentration of phenolic compounds. Further fractionation and identification of the bioactive constituents are needed to better understand the mechanisms underlying the observed antimicrobial activity and to support future valorization of *S. verticillata* roots as a source of natural antimicrobial compounds.

Cyclooxygenases (COX-1 and COX-2) are key enzymes responsible for the conversion of arachidonic acid into prostaglandins, which play central roles in inflammatory processes. COX-1 is constitutively expressed in most tissues and participates in physiological functions such as gastric mucosal protection and platelet aggregation, whereas COX-2 is mainly induced during inflammation and is responsible for the increased production of pro-inflammatory prostaglandins [35]. In the present study, all *S. verticillata* root extracts inhibited both COX isoforms, with a more pronounced inhibitory effect toward COX-1 under the applied experimental conditions. ME and UE exhibited significantly higher COX-1 inhibition than EE (*p* < 0.05), while their activities were comparable to each other and did not differ significantly from that of indomethacin (*p* > 0.05). In contrast, EE exhibited the highest COX-2 inhibition among the investigated extracts. These results demonstrate the anti-inflammatory potential of *S. verticillata* root extracts, with a more pronounced inhibitory effect toward COX-1 under the applied experimental conditions. Comparable anti-inflammatory activity has been reported for other members of the Lamiaceae family. Mićović et al. [35] investigated methanolic extracts of *Hyssopus officinalis* and observed COX-2 inhibition ranging from 54.04 to 63.04% at 20 μg mL^−1^ using the same Cayman assay, with celecoxib producing 61.60% inhibition under identical experimental conditions. The authors suggested that rosmarinic acid and chlorogenic acid contributed substantially to the observed activity while emphasizing that synergistic interactions among phenolic constituents were likely responsible for the overall anti-inflammatory effect. Similarly, Gutierrez-Albanchez et al. [36] reported that *Salvia rosmarinus* extracts inhibited both COX-1 and COX-2 with IC_50_ values of approximately 14 μg mL^−1^. The authors further demonstrated that extracts containing higher levels of rosmarinic acid inhibited both enzymes at lower concentrations, suggesting that rosmarinic acid may contribute to the anti-inflammatory activity of the extracts. In the present study, however, differences in the rosmarinic acid content of the *S. verticillata* root extracts were not directly reflected in their COX inhibitory activity. Although UE contained the highest levels of total phenolics and rosmarinic acid, ME and UE exhibited comparable COX-1 and COX-2 inhibitory activities (*p* > 0.05), whereas EE showed significantly lower COX-1 but significantly higher COX-2 inhibition than ME and UE (*p* < 0.05). Furthermore, despite its considerably lower rosmarinic acid content, EE exhibited the highest COX-2 inhibition among the extracts. These findings indicate that rosmarinic acid content alone does not determine the COX inhibitory activity of the extracts. This interpretation is further supported by the activity of rosmarinic acid tested as an individual compound. Although its COX-1 inhibitory activity did not differ significantly from any of the three extracts (*p* > 0.05), rosmarinic acid alone exhibited significantly lower COX-2 inhibition than all three crude extracts (*p* < 0.05). This finding suggests that other constituents of the extracts, as well as potential additive or synergistic interactions among rosmarinic acid, other phenolic acids, flavonoids, and additional constituents extracted by different extraction techniques, may contribute to the overall COX inhibitory activity [35,36]. Beyond direct enzyme inhibition, rosmarinic acid has also been shown to modulate inflammatory signaling pathways in previous studies. Scheckel et al. [37] demonstrated that rosmarinic acid suppresses COX-2 expression by inhibiting AP-1-dependent transcription through reduced ERK1/2 activation and decreased binding of c-Jun and c-Fos to the COX-2 promoter in both cancer and non-malignant cell lines. These observations suggest that, besides direct enzyme inhibition, rosmarinic acid may also modulate inflammatory responses through regulation of signaling pathways controlling COX-2 expression. Taken together, the present findings suggest that the extraction method influences COX inhibitory activity not only by affecting the abundance of rosmarinic acid but also by modifying the overall phytochemical composition of the extracts. Further molecular and in vivo studies are required to clarify the individual and synergistic contributions of the identified constituents to the observed COX inhibitory activity.

Based on the phytochemical characterization and biological activities of the investigated extracts, UE and EE were selected for cytotoxicity evaluation, as they represented the two extracts with the most distinct phytochemical profiles. Compared with EE, the UE extract showed cytotoxic activity against a broader range of cell lines, although the observed IC_50_ values (155–191 µg mL^−1^) indicate moderate cytotoxic activity. These differences suggest that optimization of the extraction process influenced not only the recovery of bioactive constituents but also the biological properties of the resulting extracts. Previous studies have shown that the cytotoxic activity of *Salvia* extracts depends on the plant material, extraction solvent, and chemical composition. Katanić Stanković et al. [8] reported that a methanolic extract of the aerial parts of *S. verticillata*, although rich in rosmarinic acid and other phenolic compounds, did not exhibit detectable cytotoxicity toward HaCaT keratinocytes or Balb/c-3T3 fibroblasts at concentrations up to 50 µg mL^−1^. In the present study, the UE extract reduced the viability of HaCaT cells, suggesting that differences in the plant organ used and extraction procedure may substantially influence the cytotoxic response. In contrast, Barjaktarević et al. [33] reported considerably stronger cytotoxic activity of petroleum ether and chloroform root extracts against MDA-MB-231 and HCT-116 cells, with IC_50_ values ranging from 30.90 to 105.08 µg mL^−1^. Although direct comparison is limited by differences in extract polarity and the cell lines investigated, both studies indicate that *S. verticillata* roots contain constituents with measurable cytotoxic potential. A concentration-dependent cytotoxic effect has also been reported for the essential oil of *S. verticillata* against HT-29, Caco-2, and T-47D cell lines and was attributed mainly to its high content of sesquiterpenes and other volatile constituents [34]. Collectively, these studies suggest that the cytotoxic potential of *S. verticillata* depends strongly on the extracted fraction. The broader cytotoxic activity of the UE extract may reflect the enrichment of multiple bioactive constituents achieved under the optimized extraction conditions. Although RA was the predominant phenolic compound in the UE extract, the observed cytotoxic activity is unlikely to be attributed solely to this metabolite but rather to interactions among several phytochemical constituents [7,8]. An additional finding of this study was that no IC_50_ value was reached for BALB/c-3T3 fibroblasts for either investigated extract within the tested concentration range. A431 cells were affected by both extracts, with IC_50_ values of 155 and 183 µg mL^−1^ for UE and EE, respectively. However, the similar IC_50_ values obtained for A431 (155 µg mL^−1^) and normal HaCaT cells (160 µg mL^−1^) following UE treatment do not support preferential cytotoxicity of this extract toward the A431 cancer cell line. Therefore, the observed effects should be interpreted as moderate cytotoxic activity rather than evidence of selective toxicity toward cancer cells. Further studies involving a broader panel of normal and tumor cell lines, together with investigations of apoptosis, cell-cycle arrest, and other mechanisms underlying the observed reduction in cell viability, are needed to further characterize the cytotoxic properties of these extracts. These findings emphasize that extraction conditions influence not only the phytochemical composition but also the cytotoxic profile of *S. verticillata* root extracts, highlighting the importance of extraction optimization when evaluating their biological potential.

## 4. Materials and Methods

### 4.1. Chemicals and Reagents

Rosmarinic acid standard, indomethacin, celecoxib, arachidonic acid, adrenaline bitartrate, hematin, ethylenediaminetetraacetic acid disodium salt (EDTA-Na_2_, Titriplex^®^ III), tris(hydroxymethyl)aminomethane (TRIS), 2,2-diphenyl-1-picrylhydrazyl (DPPH), 2,2′-azino-bis(3-ethylbenzothiazoline-6-sulfonic acid) (ABTS), Tyrosinase from mushroom (lyophilized powder, ≥1000 units/mg solid), 3,4-Dihydroxy-L-phenylalanine (L-DOPA), and all other chemicals and reagents used for phytochemical and biological assays were purchased from Sigma–Aldrich (Deisenhofen, Germany) or Acros Organics (Fair Lawn, NJ, USA). HPLC-grade acetonitrile and trifluoroacetic acid were obtained from Carl Roth (Karlsruhe, Germany), while ultrapure water was prepared using a Milli-Q purification system (Millipore, Bedford, MA, USA). Nutrient agar (NA), Mueller–Hinton broth (MHB), Sabouraud dextrose agar (SDA), and Sabouraud dextrose broth (SDB) used for antimicrobial assays were purchased from the Institute of Virology, Vaccines and Sera “Torlak” (Belgrade, Serbia). COX-1 (ovine; Cayman Chemical, Cat. No. 60100) and COX-2 (human recombinant; Cayman Chemical, Cat. No. 60122) enzymes used for the anti-inflammatory assay were obtained from Cayman Chemical (Ann Arbor, MI, USA).

### 4.2. Plant Material

*Salvia verticillata* L. roots were collected in June 2025, during the flowering stage, in the village of Borač (43.9646° N, 20.6034° E), near Kragujevac, Šumadija, central Serbia, at an altitude of approximately 350 m a.s.l. (Republic Hydrometeorological Service of Serbia, Borač hydrological station: https://www.hidmet.gov.rs/latin/hidrologija/izvestajne/bezprognoza.php?hm_id=47156; accessed on 10 August 2026). The broader Gruža–Kragujevac region is characterized by a temperate continental climate, with warm summers and cold winters; long-term climatological data for the nearby Kragujevac meteorological station indicate a mean annual temperature of approximately 11 °C, while annual precipitation in the region is approximately 550–650 mm, with a greater proportion occurring during the warmer part of the year. A voucher specimen (No. 146/26) was deposited in the Herbarium of the Department of Biology and Ecology, Faculty of Science, University of Kragujevac (Kragujevac, Serbia). Taxonomic and botanical identification was confirmed by Dr. Milan S. Stanković. Following collection, the roots were separated from the aerial parts, dried at 25 ± 2 °C and a relative humidity (RH) of 50–60% in a dark, well-ventilated area, and stored in paper bags until further analysis.

### 4.3. Extraction Procedures

#### 4.3.1. Ethnopharmacological Extraction (EE)

Dried and powdered *S. verticillata* roots (50 g) were extracted with 250 mL of 96% ethanol at 25 ± 2 °C and 50–60% RH for 3 weeks (21 days) with occasional stirring, following the traditional ethnopharmacological extraction procedure. The extract was filtered through filter paper and concentrated under reduced pressure using a rotary evaporator (RV 10 basic, IKA, Staufen, Germany) to obtain the crude extract. The obtained extract was stored at 4 °C until further analysis.

#### 4.3.2. Conventional Maceration (ME)

Dried and powdered *S. verticillata* roots (50 g) were extracted by maceration with 250 mL of 96% ethanol at 25 ± 2 °C and 50–60% RH in the dark. After 24 h, the extract was filtered through filter paper, and the plant residue was re-extracted with a fresh portion of 250 mL of 96% ethanol under the same conditions. The filtration procedure was repeated after another 24 h, and the residue was extracted once more with 250 mL of 96% ethanol. After a further 24 h, the extract was filtered through filter paper, and the filtrates were combined. The combined filtrates were concentrated under reduced pressure using a rotary evaporator (RV 10 basic, IKA, Staufen, Germany) and stored at 4 °C until analysis.

#### 4.3.3. Ultrasonic-Assisted Extraction (UE)

Thirty samples (1.0 g each) of dried and powdered *S. verticillata* roots were extracted according to the experimental conditions defined by the Central Composite Design (CCD). The extraction variables included ethanol concentration (20–100%, *v*/*v*), solvent-to-solid ratio (10–40 mL g^−1^), extraction time (10–30 min), and extraction temperature (25–75 °C). Ultrasound-assisted extraction was performed in an ultrasonic bath (Bandelin Sonorex RK 52 H, Bandelin electronic GmbH & Co. KG, Berlin, Germany). After extraction, the samples were filtered through filter paper, and the filtrates were used directly for subsequent analyses. Following the optimization of extraction conditions, a larger batch of the optimized ultrasound-assisted extract was prepared using 50 g of dried and powdered *S. verticillata* roots under the optimal extraction conditions described in Section 2.1.4. This extract was used for all subsequent phytochemical and biological analyses.

### 4.4. Experimental Design and Optimization of Ultrasound-Assisted Extraction

Ultrasound-assisted extraction (UAE) was optimized using Response Surface Methodology (RSM) based on a Central Composite Design (CCD) generated in Design-Expert^®^ software, Version 13 (Stat-Ease Inc., Minneapolis, MN, USA). The effects of ethanol concentration, solvent-to-solid ratio, extraction time, and extraction temperature on total phenolic content (TPC) and rosmarinic acid (RA) concentration were investigated. The experimental data were analyzed by analysis of variance (ANOVA), and the adequacy of the fitted models was evaluated using the coefficient of determination (R^2^), adjusted and predicted R^2^ values, lack-of-fit test, coefficient of variation (CV), and adequate precision. Numerical optimization was performed using the desirability function approach to simultaneously maximize TPC and RA concentration while maintaining all extraction variables within the investigated experimental ranges.

### 4.5. Determination of Total Phenolic Content

The total phenolic content (TPC) of the extracts was determined using the Folin–Ciocalteu colorimetric method according to Singleton et al. [38] with slight modifications. Briefly, 0.5 mL of the extract solution (0.5 mg mL^−1^) was mixed with 2.5 mL of ten-fold diluted Folin–Ciocalteu reagent, followed by the addition of 2.0 mL of 7.5% (*w*/*v*) sodium bicarbonate solution. After incubation for 15 min at 45 °C, the absorbance was measured at 765 nm using double beam spectrophotometer Halo DB-20S (Dynamica GmbH, Dietikon, Switzerland). Gallic acid was used as the reference standard, and the results were calculated from the calibration curve and expressed as milligrams of gallic acid equivalents per gram of dry extract (mg GAE g^−1^ d.e.).

### 4.6. Determination of Total Flavonoid Content

The total flavonoid content (TFC) was determined using the aluminum chloride colorimetric method according to Brighente et al. [39]. Briefly, the extracts were dissolved in methanol to a concentration of 0.5 mg mL^−1^. An aliquot of 0.5 mL of the extract solution was mixed with an equal volume of 2% (*w*/*v*) aluminum chloride solution prepared in methanol. The reaction mixture was incubated for 1 h at 25 ± 2 °C and 50–60% RH in the dark, after which the absorbance was measured at 415 nm. A calibration curve was prepared using rutin as the reference standard, and the total flavonoid content was expressed as milligrams of rutin equivalents per gram of dry extract (mg RUE g^−1^ d.e.).

### 4.7. Determination of Total Phenolic Acids

The total phenolic acid content (TPA) was determined according to the method described in the Polish Pharmacopoeia, as reported by Matkowski et al. [40]. Briefly, 1.0 mL of the extract solution (1 mg mL^−1^) was mixed with 5.0 mL of distilled water, followed by the sequential addition of 1.0 mL of 0.1 M hydrochloric acid, 1.0 mL of Arnow reagent (10% (*w*/*v*) sodium nitrite and 10% (*w*/*v*) sodium molybdate), and 1.0 mL of 1 M sodium hydroxide solution. The reaction mixture was diluted to a final volume of 10 mL with distilled water, and the absorbance was measured immediately at 490 nm. Caffeic acid was used as the reference standard, and the total phenolic acid content was expressed as milligrams of caffeic acid equivalents per gram of dry extract (mg CAE g^−1^ d.e.).

### 4.8. HPLC Analysis of Rosmarinic Acid

Rosmarinic acid was quantified using a Shimadzu Prominence HPLC system (Kyoto, Japan) equipped with a CBM-20A system controller, an LC-20AD solvent delivery pump with an online degasser (DGU-20A5), a CPO-20AC column oven, and an SPD-M20A photodiode array (PDA) detector. Instrument control, data acquisition, and chromatographic processing were performed using LC solution software (version 1.24 SP1). Chromatographic separation was achieved on a Luna C18 column (250 × 4.6 mm, 5 μm; Phenomenex, Torrance, CA, USA). The mobile phase consisted of solvent A (water containing 0.1% trifluoroacetic acid) and solvent B (acetonitrile containing 0.1% trifluoroacetic acid). Elution was performed at a flow rate of 1.0 mL min^−1^ using the following gradient program: 5% B (0–1 min), 5–10% B (1–5 min), 10–80% B (5–25 min), 100% B (25–27 min), followed by re-equilibration to 5% B (27–30 min). The injection volume was 20 μL, and chromatograms were monitored at 280, 325, and 360 nm. Rosmarinic acid was identified by comparing its retention time and UV–Vis absorption spectrum with those of the reference standard. Quantification was performed at 325 nm by the external standard method using a calibration curve, and the results were expressed as mg g^−1^ dry extract. Each sample was analyzed in triplicate.

### 4.9. Antioxidant Activity

#### 4.9.1. Total Antioxidant Capacity

The total antioxidant capacity (TAC) of the extracts was determined using the phosphomolybdenum assay according to Prieto et al. [41]. Briefly, 0.3 mL of the extract solution was mixed with 3.0 mL of phosphomolybdenum reagent containing 0.6 M sulfuric acid, 28 mM sodium phosphate, and 4 mM ammonium molybdate. The reaction mixture was incubated at 95 °C for 90 min, allowed to cool to 25 ± 2 °C, and the absorbance was measured at 695 nm. The total antioxidant capacity was expressed as milligrams of ascorbic acid equivalents per gram of dry extract (mg AA g^−1^ d.e.).

#### 4.9.2. Reducing Power

The reducing power of the extracts was determined according to the method described by Oyaizu [42]. Briefly, 1.0 mL of the extract solution (0.1 mg mL^−1^) was mixed with 1.0 mL of 0.2 M sodium phosphate buffer (pH 6.6) and 1.0 mL of 1% (*w*/*v*) potassium ferricyanide solution. The reaction mixture was incubated at 50 °C for 20 min, followed by the addition of 1.0 mL of 10% (*w*/*v*) trichloroacetic acid. After centrifugation at 2000 rpm for 8 min using a D1012UA high-speed microcentrifuge (DLAB Scientific Co., Ltd., Beijing, China), the resulting supernatant was mixed with 0.8 mL of 0.1% ferric chloride (FeCl_3_), and the absorbance was measured at 700 nm. The reducing power was expressed as milligrams of Trolox equivalents per gram of dry extract (mg Trolox g^−1^ d.e.).

#### 4.9.3. DPPH Radical Scavenging Activity

The free radical scavenging activity of the extracts was evaluated using the DPPH assay according to Kumarasamy et al. [43]. Briefly, 1.0 mL of the extract solution or positive control was mixed with 1.0 mL of DPPH solution (80 μg mL^−1^) and incubated in the dark at 25 ± 2 °C and 50–60% RH for 30 min. The absorbance was measured at 517 nm. Gallic acid and ascorbic acid were used as positive controls. The radical scavenging activity was expressed as the IC_50_ value (μg mL^−1^), defined as the concentration required to scavenge 50% of DPPH radicals.

#### 4.9.4. ABTS Radical Scavenging Activity

The ABTS radical scavenging activity of the extracts was determined according to the method described by Re et al. [44]. The ABTS^•+^ radical cation was generated by reacting 7 mM ABTS solution with 2.45 mM potassium persulfate, and the mixture was allowed to stand in the dark at 25 ± 2 °C and 50–60% RH for 16 h. Prior to analysis, the ABTS^•+^ solution was diluted with methanol to obtain an absorbance of 0.70 ± 0.02 at 734 nm. Briefly, 200 μL of serially diluted extract solution or positive control was mixed with 1.8 mL of the ABTS^•+^ solution and incubated at 30 °C for 30 min. The absorbance was measured at 734 nm. Gallic acid was used as a positive control. The radical scavenging activity was expressed as the IC_50_ value (μg mL^−1^), corresponding to the concentration required to scavenge 50% of ABTS^•+^ radicals.

### 4.10. Determination of Tyrosinase Inhibitory Activity

Tyrosinase inhibitory activity was evaluated spectrophotometrically using a modified dopachrome assay, as previously described by Pohntadavit et al. [45]. Mushroom tyrosinase was dissolved in 50 mM phosphate buffer (pH 6.8) to obtain a final concentration of 100 U mL^−1^. Freshly prepared 3,4-dihydroxy-L-phenylalanine (L-DOPA) was dissolved in the same buffer at a concentration of 10 mM. Serial dilutions of the *S. verticillata* extracts were prepared in phosphate buffer. The reaction mixture consisted of 160 µL of sample solution, 480 µL of phosphate buffer, and 80 µL of tyrosinase solution. After pre-incubation of the reaction mixture for 10 min at 37 °C, the reaction was initiated by adding 80 µL of 10 mM L-DOPA, resulting in a final substrate concentration of 1 mM. The formation of dopachrome was monitored at 475 nm using Halo DB-20S double beam spectrophotometer (Dynamica GmbH, Dietikon, Switzerland) by recording the absorbance every 30 s for 10 min. The initial reaction rate (ΔA_475_ min^−1^) was calculated from the linear portion of the kinetic curve. Tyrosinase inhibition was calculated according to the following equation:

$$\mathrm{Inhibition}\;(\%)=100\times \left(1-\frac{v_{sample}}{v_{control}}\right)$$

where $v_{\mathrm{sample}}$ and $v_{\mathrm{control}}$ represent the initial reaction rates, calculated from the linear portion of the kinetic curves, measured in the presence and absence of the extract, respectively. IC_50_ values were determined by nonlinear regression analysis of inhibition percentage versus extract concentration using OriginPro 8 (OriginLab Corporation, Northampton, MA, USA).

### 4.11. Determination of Antimicrobial Activity

#### 4.11.1. Microorganisms

The antimicrobial potential of *S. verticillata* root extracts was assessed using both reference (ATCC) and clinically isolated microbial strains. A total of twenty microorganisms were included in the study, comprising eleven bacteria and nine fungal species. The tested bacterial strains were *Escherichia coli* (ATCC 25922), *Staphylococcus aureus* (ATCC 25923), *Bacillus subtilis* (ATCC 6633), *Pseudomonas aeruginosa* (ATCC 10145), *Micrococcus lysodeikticus* (ATCC 4698), *Salmonella typhimurium* (ATCC 14028), *Enterococcus faecalis* (ATCC 29212), *Klebsiella pneumoniae* (ATCC 70063), *Staphylococcus epidermidis* (ATCC 12228), *Bacillus cereus* (ATCC 10876), and *Salmonella enteritidis* (ATCC 13076). The fungal panel included *Candida albicans* (ATCC 10259), *Aspergillus brasiliensis* (ATCC 16404), *Fusarium oxysporum* (FSB 91), *Alternaria alternata* (FSB 51), *Aureobasidium pullulans* (FSB 61), *Trichoderma harzianum* (FSB 12), *Trichoderma longibrachiatum* (FSB 13), *Penicillium canescens* (FSB 24), and *Doratomyces stemonitis* (FSB 41).

All microorganisms were sourced from the Institute of Public Health Kragujevac and the Laboratory for Microbiology, Department of Biology, Faculty of Science, University of Kragujevac (Serbia). Prior to testing, bacterial strains were cultured on nutrient agar, *Candida albicans* on Sabouraud dextrose agar (both at 37 °C for 24 h), and mold strains on potato dextrose agar (PDA) at 28 °C for 72 h.

#### 4.11.2. Microdilution Method

The minimum inhibitory concentrations (MICs) of the tested extracts were determined using the broth microdilution method, following the procedure described by Sarker et al. [46], with minor modifications. Antibacterial activity was evaluated in Mueller–Hinton broth (MHB), while antifungal activity was assessed in Sabouraud dextrose broth (SDB) using sterile 96-well microtiter plates. Serial twofold dilutions of the extracts were prepared in the appropriate media, starting from an initial concentration of 20 mg/mL. For antibacterial assays, each well contained extract dilutions in MHB, followed by the addition of bacterial inoculum (approximately 1.0 × 10^6^ CFU/mL) and resazurin solution as a growth indicator. For antifungal assays, extract dilutions in SDB were inoculated with fungal suspensions (approximately 1.0 × 10^4^ CFU/mL). Reference antimicrobial agents (Tetracycline for bacteria and Nystatin for fungi) were included as positive controls. Microbial suspensions were prepared in sterile saline according to CLSI guidelines [47,48,49]. The microplates were incubated at 37 °C for 24 h for bacterial strains and at 28 °C for 48 h for fungal strains. Bacterial growth was evaluated based on the color change in resazurin, whereas fungal growth was assessed visually. MIC values were defined as the lowest concentration of the extract that inhibited visible microbial growth, indicated by the absence of color change (bacteria) or lack of visible growth (fungi).

### 4.12. Evaluation of Anti-Inflammatory Activity

Anti-inflammatory activity was quantified using a commercially available assay kit (Enzo Life Sciences, Farmingdale, NY, USA). The assay was based on an in vitro system in which COX-1 (ovine; Cayman Chemical, 60100) and COX-2 (human recombinant; Cayman Chemical, 60122) enzymes (0.2 U/well) catalyzed the formation of prostaglandin E2 (PGE2). All experiments were performed in 96-well microplates. All extracts were tested at a concentration of 50 μg/mL. Rosmarinic acid was tested as the most abundant phenolic compound at a concentration of 10 μM. Indomethacin (purity ≥ 99%, 1.25 μM) and celecoxib (purity ≥ 98%, concentration 8.8 µM), previously dissolved in ethanol p.a. (pro analysis), were used as positive controls, respectively.

In addition to COX-1, COX-2, arachidonic acid, and the tested extracts, the reaction mixture contained adrenaline bitartrate (18 mM), hematin (100 µM), and EDTA-Na2 (Titriplex^®^ III, 1 mM) to support PGE2 production. EDTA-Na2 (Titriplex^®^ III) was prepared in 0.1 M TRIS/HCl buffer. Following incubation, the enzymatic reaction was terminated according to the manufacturer’s protocol. The resulting yellow color was measured in an ELISA microplate using a Hidex microplate reader (Hidex Oy, Turku, Finland) at a wavelength of 405 nm.

### 4.13. Evaluation of Cytotoxic Activity

Human HaCaT keratinocytes were obtained from Innoprot (Derio, Spain), whereas human A431 epidermoid carcinoma cells and murine BALB/c-3T3 and SVT2 fibroblasts were purchased from the American Type Culture Collection (ATCC, Manassas, VA, USA). Cells were cultured in Dulbecco’s Modified Eagle’s Medium, supplemented with 10% fetal bovine serum, 2 mM L-glutamine and antibiotics (streptomycin and penicillin) in a 5% CO_2_ humidified atmosphere at 37 °C as previously described by Imbimbo et al. [50]. The biocompatibility of UE and EE extracts was evaluated by the MTT assay. Cells were seeded in a 96-well plate at a density of 5 × 10^3^ cell/well and incubated with increasing concentrations of the extracts (1–200 μg mL^−1^). After 72 h incubation, cell viability was assessed by the MTT assay [51]. Cell survival was expressed as the percentage of viable cells in the presence of the molecules compared to untreated control cells. Cells treated with the corresponding volume of extraction solvent served as vehicle controls. All experiments were performed in three independent experiments, each carried out in triplicate.

### 4.14. Statistical Analysis

All experiments were performed in triplicate, and the results are presented as the mean ± standard deviation (SD). The IC_50_ values for DPPH and ABTS radical scavenging assays were calculated by fitting the inhibition curves using a sigmoidal dose–response model in OriginPro 8 (OriginLab, Northampton, MA, USA). The IC_50_ value was defined as the concentration of the extract required to inhibit 50% of free radicals. Statistical analyses were performed using SPSS Statistics version 13.0 (IBM Corp., Armonk, NY, USA). Differences between mean values were evaluated by one-way analysis of variance (ANOVA), and statistical significance was accepted at *p* < 0.05.

## 5. Conclusions

This study provides the first comprehensive evaluation of *Salvia verticillata* L. roots by integrating extraction optimization, phytochemical characterization, and biological activity assessment. Response surface methodology proved to be an effective approach for optimizing ultrasound-assisted extraction, providing conditions for the rapid and efficient recovery of phenolic compounds, particularly rosmarinic acid, from *S. verticillata* roots. The optimized ultrasound-assisted extract generally exhibited higher antioxidant activity, while all three extracts showed notable tyrosinase inhibitory activity. The ethnopharmacological extract displayed more pronounced antibacterial activity and higher COX-2 inhibition, whereas cytotoxicity evaluation revealed moderate activity of UE against A431, SVT2, and HaCaT cells and of EE against A431 cells within the tested concentration range. All extracts demonstrated substantial COX-1 inhibitory activity, moderate antibacterial activity, particularly against Gram-positive bacteria, and limited antifungal activity. Rosmarinic acid was identified as the predominant phenolic constituent in all extracts. Nevertheless, the observed biological activities were not consistently associated with its concentration, as particularly evident from the COX inhibition results, suggesting that other phenolic constituents and their interactions also contribute to the overall bioactivity. Importantly, the optimized ultrasound-assisted extract exhibited biological activities comparable to, and in some cases more pronounced than, those of the ethnopharmacological extract, indicating that ultrasound-assisted extraction represents a rapid and efficient alternative to conventional extraction while substantially reducing extraction time. These findings highlight the potential of *S. verticillata* roots as a valuable source of bioactive compounds and support the use of ultrasound-assisted extraction as a rapid and efficient method for producing extracts intended for pharmaceutical, nutraceutical, food, and cosmetic applications. Further studies involving comprehensive metabolite profiling, identification of additional bioactive constituents, and in vivo validation are needed to support the future development and utilization of *S. verticillata* root-derived products.

## Figures and Tables

**Table 1:** Experimental design matrix (CCD) and experimental response values for total phenolic content (TPC) and rosmarinic acid (RA).

Std	Run	A: EtOH (%)	B: Solvent to Solid (mL g^−1^)	C: Time (min)	D: Temperature (°C)	TPC (mg GAE g^−1^ DW)	RA (μg g^−1^ DW)
7	1	20	40	30	25	39.9857	10,955.7
24	2	60	25	20	75	25.512	31,050.9
4	3	100	40	10	25	21.3958	5892.61
23	4	60	25	20	25	22.4834	30,200.7
6	5	100	10	30	25	14.4381	11,090.2
8	6	100	40	30	25	29.9541	13,773
22	7	60	25	30	50	29.5721	18,547.7
18	8	100	25	20	50	26.2608	14,608.8
10	9	100	10	10	75	14.6409	13,610.6
11	10	20	40	10	75	46.4029	19,440.4
27	11	60	25	20	50	32.4962	28,444.8
13	12	20	10	30	75	6.04177	20,225.9
28	13	60	25	20	50	29.1136	18,205.7
25	14	60	25	20	50	30.54	16,347.1
5	15	20	10	30	25	5.41009	12,656.5
29	16	60	25	20	50	31.2532	19,333.8
12	17	100	40	10	75	30.6877	17,707.4
17	18	20	25	20	50	31.1004	10,578.7
30	19	60	25	20	50	33.189	15,802.5
14	20	100	10	30	75	9.93989	17,565.4
21	21	60	25	10	50	28.5023	17,100.1
26	22	60	25	20	50	32.5003	14,325.3
3	23	20	40	10	25	34.2679	10,743.6
9	24	20	10	10	75	14.9516	16,295.8
15	25	20	40	30	75	58.3225	35,230.1
1	26	20	10	10	25	5.91951	9340.7
16	27	100	40	30	75	46.2557	32,945.5
19	28	60	10	20	50	33.1223	14,063.6
2	29	100	10	10	25	12.1549	8589.81
20	30	60	40	20	50	56.3627	26,469.6

Std—standard order of the experimental design; CCD—Central Composite Design; EtOH—ethanol; TPC—total phenolic content; RA—rosmarinic acid; GAE—gallic acid equivalents; DW—dry weight of plant material.

**Table 2:** Regression equations and statistical parameters of the developed models for total phenolic content (TPC) and rosmarinic acid (RA).

Parameter	TPC (mg GAE g^−1^ DW)	RA (µg g^−1^ DW)
Model type	Quadratic	Reduced quadratic
Model F-value	53.42	9.29
Model *p*-value	<0.0001	<0.0001
Lack of fit F-value	4.10	0.66
Lack of fit *p*-value	0.0665	0.7596
R^2^	0.9803	0.7473
Adjusted R^2^	0.9620	0.6669
Predicted R^2^	0.8832	0.5271
Adeq. Precision	28.18	10.92
C.V. (%)	9.57	24.66
Regression equations in terms of coded factors		
TPC	TPC = 32.57 − 2.04A + 13.72B + 1.72C + 3.71D − 4.35AB + 0.8431AC − 1.03AD + 3.35BC + 3.03BD − 0.1358CD − 4.94A^2^ + 11.13B^2^ − 4.58C^2^ − 9.62D^2^	
RA	RA = 19,582.44 − 538.00A + 2762.20B + 3014.95C + 5046.07D + 2371.12BD − 10,580.79A^2^ + 7451.31D^2^	

TPC—total phenolic content; RA—rosmarinic acid; GAE—gallic acid equivalents; DW—dry weight of plant material. A—ethanol concentration; B—solvent-to-solid ratio; C—extraction time; D—extraction temperature. Regression equations are expressed in terms of coded factors.

**Table 3:** Optimization criteria used for numerical optimization.

Name	Goal	Lower Limit	Upper Limit	Lower Weight	Upper Weight	Importance
A: EtOH	is in range	20	100	1	1	3
B: Solvent to solid	is in range	10	40	1	1	3
C: Time	is in range	10	30	1	1	3
D: Temperature	is in range	25	75	1	1	3
Total phenolic content	maximize	5.41009	58.3225	1	1	5
Rosmarinic acid	maximize	5892.61	35,230.1	1	1	3

**Table 4:** Quantitative determination of total phenolic content (TPC), total flavonoid content (TFC), total phenolic acid content (TPA), and rosmarinic acid (RA) in *S. verticillata* root extracts.

Extracts	TPC (mg GAE g^−1^ d.e.)	TFC (mg RUE g^−1^ d.e.)	TPA (mg CAE g^−1^ d.e.)	RA (mg g^−1^ d.e.)
EE	304.2 ± 1.6 ^a^	47.2 ± 2.1 ^a^	22.4 ± 1.2 ^a^	129.26 ± 0.08 ^b^
ME	207.2 ± 19.9 ^b^	23.8 ± 1.2 ^c^	14.5 ± 0.1 ^b^	96.67 ± 0.05 ^c^
UE	313.9 ± 22.1 ^a^	34.2 ± 1.8 ^b^	21.1 ± 0.4 ^a^	137.47 ± 0.09 ^a^

EE—ethnopharmacological extraction; ME—maceration; UE—optimized ultrasound-assisted extraction; GAE—gallic acid equivalents; RUE—rutin equivalents; CAE—caffeic acid equivalents; d.e.—dry extract. Different superscript letters within the same column indicate statistically significant differences (*p* < 0.05).

**Table 5:** Antioxidant activity of *S. verticillata* root extracts determined by total antioxidant capacity (TAC), reducing power (RP), DPPH, and ABTS assays.

Extracts/ Standards	Total Antioxidant Capacity (mg AA g^−1^ d.e.)	Reducing Power (mg Trolox g^−1^ d.e.)	IC_50_ (µg mL^−1^)	
			DPPH^●^ Scavenging Activity	ABTS^+^ Scavenging Activity
EE	1368.9 ± 26.1 ^a^	546.2 ± 6.4 ^b^	17.4 ± 0.2 ^c^	67.9 ± 4.4 ^b^
ME	948.7 ± 7.1 ^c^	342.2 ± 11.2 ^c^	35.6 ± 0.3 ^e^	94.6 ± 9.2 ^c^
UE	1256.7 ± 16.3 ^b^	957.3 ± 3.1 ^a^	10.2 ± 0.1 ^b^	70.4 ± 5.1 ^b^
GA	-	-	1.5 ± 0.1 ^a^	2.5 ± 0.2 ^a^
AA	-	-	20.6 ± 0.6 ^d^	-

EE—ethnopharmacological extraction; ME—maceration; UE—optimized ultrasound-assisted extraction; GA—gallic acid; AA—ascorbic acid; d.e.—dry extract. Different superscript letters within the same column indicate statistically significant differences (*p* < 0.05).

**Table 6:** Minimum inhibitory concentration (MIC) values of *S. verticillata* root extracts against bacterial and fungal strains.

Microorganisms	MIC (mg mL^−1^)			MIC (mg mL^−1^)
	EE	ME	UE	Standard
Bacteria				Tetracycline
*K. pneumoniae*	10	20	20	1.25 × 10^−3^
*E. coli*	20	20	20	6.25 × 10^−4^
*P. aeruginosa*	20	20	20	0.01
*E. faecalis*	10	20	10	0.01
*B. subtilis*	<0.156	<0.156	<0.156	0.01
*B. cereus*	5	20	20	1.25 × 10^−3^
*S. aureus*	0.156	0.3125	0.3125	6.25 × 10^−4^
*S. epidermidis*	20	20	20	1.25 × 10^−3^
*M. lysodeikticus*	20	20	20	<3.125 × 10^−4^
*S. enteritidis*	20	20	20	6.25 × 10^−4^
*S. typhimurium*	1.25	2.5	2.5	2.5 × 10^−3^
Fungi				Nystatin
*C. albicans*	20	20	10	0.01
*A. brasiliensis*	20	20	20	0.02
*T. harzianum*	20	20	20	0.02
*T. longibrachiatum*	20	20	20	0.01
*F. oxysporum*	20	20	20	0.02
*A. alternata*	>20	>20	>20	0.02
*D. stemonitis*	20	20	20	0.01
*P. cyclopium*	20	20	20	0.01
*P. canescens*	20	20	20	0.01

MIC—Minimum inhibitory concentration; EE—ethnopharmacological extraction; ME—maceration; UE—optimized ultrasound-assisted extraction.

**Table 7:** The results of the ability of *S. verticillata* ethanol extracts (EE, ME, and UE) to inhibit the activity of COX-1 and COX-2 enzymes (% inhibition).

Sample	COX-1 Inhibition (%)	COX-2 Inhibition (%)
EE	71.8 ± 0.9 ^c^	56.5 ± 2.1 ^b^
ME	80.2 ± 1.75 ^a,b^	42.8 ± 2.5 ^c^
UE	79.8 ± 0.2 ^a,b^	42.9 ± 5.6 ^c^
Rosmarinic acid	73.8 ± 5.3 ^b,c^	19.4 ± 3.0 ^d^
Indomethacin	85.1 ± 1.0 ^a^	/
Celecoxib	/	83.6 ± 0.4 ^a^

The percent of inhibition of COX enzyme activity obtained in two independent experiments (the mean value ± SD). Different superscript letters within the same column indicate statistically significant differences (*p* < 0.05).

**Table 8:** IC_50_ values (µg mL^−1^) of *S. verticillata* root extracts determined by the MTT assay on different cell lines after 72 h incubation.

Cell Line	EE	UE
HaCaT	N.D.	160 ± 6
Balb/c-3T3	N.D.	N.D.
A431	183 ± 1	155 ± 13
SVT2	N.D.	191 ± 10

EE—ethnopharmacological extraction; UE—optimized ultrasound-assisted extraction; N.D.—not determined within the tested concentration range.

## Data Availability

The original contributions presented in the study are included in the article. Further inquiries can be directed to the corresponding authors.

## Abbreviations

The following abbreviations are used in this manuscript:

AA	Ascorbic acid
ABTS	2,2′-azino-bis(3-ethylbenzothiazoline-6-sulfonic acid)
ANOVA	Analysis of variance
ATCC	American Type Culture Collection
CAE	Caffeic acid equivalents
CCD	Central composite design
CFU	Colony-forming units
CLSI	Clinical and Laboratory Standards Institute
COX-1	Cyclooxygenase-1
COX-2	Cyclooxygenase-2
d.e.	Dry extract
DPPH	2,2-Diphenyl-1-picrylhydrazyl
DW	Dry weight of plant material
EDTA-Na_2_	Ethylenediaminetetraacetic acid disodium salt
EE	Ethnopharmacological extraction
ELISA	Enzyme-linked immunosorbent assay
GA	Gallic acid
GAE	Gallic acid equivalents
HPLC	High-performance liquid chromatography
IC_50_	Half-maximal inhibitory concentration
KA	Kojic acid
L-DOPA	3,4-Dihydroxy-L-phenylalanine
ME	Maceration extraction
MHB	Mueller–Hinton broth
MIC	Minimum inhibitory concentration
MTT	3-(4,5-Dimethylthiazol-2-yl)-2,5-diphenyltetrazolium bromide
NA	Nutrient agar
PDA	Photodiode array
PGE_2_	Prostaglandin E_2_
RA	Rosmarinic acid
RP	Reducing power
RSM	Response surface methodology
RUE	Rutin equivalents
SD	Standard deviation
SDA	Sabouraud dextrose agar
SDB	Sabouraud dextrose broth
TAC	Total antioxidant capacity
TFC	Total flavonoid content
TPA	Total phenolic acid content
TPC	Total phenolic content
TRIS	Tris(hydroxymethyl)aminomethane
UE	Ultrasound-assisted extraction
